# The TGFβ Induced MicroRNAome of the Trabecular Meshwork

**DOI:** 10.3390/cells13121060

**Published:** 2024-06-19

**Authors:** Chelsey Doyle, Breedge Callaghan, Anton W. Roodnat, Lee Armstrong, Karen Lester, David A. Simpson, Sarah D. Atkinson, Carl Sheridan, Declan J. McKenna, Colin E. Willoughby

**Affiliations:** 1 Centre for Genomic Medicine, Biomedical Sciences Research Institute, Ulster University, Coleraine Campus, Coleraine BT52 1SA, UK; doyle-c29@ulster.ac.uk (C.D.); roodnat-a@ulster.ac.uk (A.W.R.); armstrong-l16@ulster.ac.uk (L.A.); s.atkinson@ulster.ac.uk (S.D.A.); dj.mckenna@ulster.ac.uk (D.J.M.); 2 Wellcome Wolfson Institute for Experimental Medicine, Queens’ University, Belfast BT9 7BL, UK; david.simpson@qub.ac.uk; 3 Department of Eye and Vision Science, Institute of Life Course and Medical Sciences, University of Liverpool, Liverpool L7 8TX, UK; c.sheridan@liverpool.ac.uk

**Keywords:** glaucoma, primary open-angle glaucoma, POAG, trabecular meshwork, intra-ocular pressure, pseudoexfoliation glaucoma, microRNA, miRNA, transforming growth factor beta, TGFβ, fibrosis, epigenetics, gene therapy, therapeutics

## Abstract

Primary open-angle glaucoma (POAG) is a progressive optic neuropathy with a complex, multifactorial aetiology. Raised intraocular pressure (IOP) is the most important clinically modifiable risk factor for POAG. All current pharmacological agents target aqueous humour dynamics to lower IOP. Newer therapeutic agents are required as some patients with POAG show a limited therapeutic response or develop ocular and systemic side effects to topical medication. Elevated IOP in POAG results from cellular and molecular changes in the trabecular meshwork driven by increased levels of transforming growth factor β (TGFβ) in the anterior segment of the eye. Understanding how TGFβ affects both the structural and functional changes in the outflow pathway and IOP is required to develop new glaucoma therapies that target the molecular pathology in the trabecular meshwork. In this study, we evaluated the effects of TGF-β1 and -β2 treatment on miRNA expression in cultured human primary trabecular meshwork cells. Our findings are presented in terms of specific miRNAs (miRNA-centric), but given miRNAs work in networks to control cellular pathways and processes, a pathway-centric view of miRNA action is also reported. Evaluating TGFβ-responsive miRNA expression in trabecular meshwork cells will further our understanding of the important pathways and changes involved in the pathogenesis of glaucoma and could lead to the development of miRNAs as new therapeutic modalities in glaucoma.

## 1. Introduction

Glaucoma is a progressive optic neuropathy with loss of retinal ganglion cells and characteristic visual field and optic nerve changes [1]. Glaucoma affects over 60 million people worldwide and the prevalence is projected to increase to over 118 million people by 2040 [2]. Primary open-angle glaucoma (POAG) forms the most prevalent subtype of glaucoma and has a complex, multifactorial aetiology. Raised intraocular pressure (IOP) is the most important clinically modifiable risk factor for POAG. IOP is generated by the conventional aqueous humour outflow pathway via the trabecular meshwork and all current pharmacological agents target aqueous humour dynamics to lower IOP [3,4].

However, some patients with POAG show a limited therapeutic response or are refractory to these pharmacological agents [5,6,7], while others have clinically significant adverse physiological impacts, such as bradycardia or bronchospasm [8]. Until the development of Rho kinase (ROCK) inhibitors, none of the topical drug treatments for POAG targeted the underlying cellular pathophysiology in the trabecular meshwork [9,10]. ROCK inhibitors pharmacologically manipulate the cytoskeleton of trabecular meshwork and Schlemm’s canal cells reducing outflow resistance and lowering IOP [9,10]. Relatively common ocular side effects like conjunctival hyperaemia have limited tolerability of ROCK inhibitors in the pharmacological management of POAG [3,4,10]. Therefore, there is a clinical need to develop novel therapies for POAG specifically targeting the molecular pathology in the trabecular meshwork to lower IOP [3,4,11].

Elevated IOP in POAG results from cellular and molecular changes in the trabecular meshwork (TM) driven by increased levels of transforming growth factor β (TGFβ) in the anterior segment of the eye [12]. TGF-β1 and -β2 result in pathogenic changes in the human trabecular meshwork (TM) cell population and phenotype, which contribute to increased IOP [13]. Various studies have identified elevated TGFβ2 levels in the aqueous humour of POAG patients [14,15,16,17,18], but the cause of these elevated levels is unclear. Elevated TGFβ2 levels have also been reported in the glaucomatous TM, indicating that increased levels of total and mature TGFβ may play an important role in the pathogenesis of POAG [19]. TGFβ2 perfusion in an anterior eye segment organ culture model resulted in elevated intra-ocular pressure (IOP) and fibrillary material accumulation in the trabecular meshwork [15,20]. In pseudoexfoliation glaucoma (XFG), an aggressive form of secondary open-angle glaucoma, both the latent and active forms of TGFβ1 are increased in the aqueous humour and extracellular matrix (ECM) [21,22,23].

The TGFβ receptor complex transmits signals via both canonical and non-canonical pathways [24,25,26]. Canonical TGFβ signalling results in Smad2/3–Smad4 complexes translocating to the nucleus where they function as transcription factors that can regulate gene expression including microRNAs (miRNAs) [27,28,29,30]. MiRNAs are small, single-stranded, noncoding RNAs which are important regulators of eukaryotic gene expression in health and disease [31]. Normally, miRNAs bind to the 3′ untranslated region (UTR) of their target mRNAs resulting in mRNA degradation or translation repression [32]. Most TGFβ signalling pathway components are known to be targeted by one or more miRNAs, and miRNA regulation of TGFβ signalling molecules influences the pathogenesis of fibrotic diseases [33]. TGFβ signalling can also regulate miRNA expression at transcriptional and post-transcriptional levels [25]. TGFβ is both cell- and context-specific in terms of its functionality. Therefore, research must focus on building our understanding of how TGFβ affects both the structural and functional changes in the outflow pathway and therefore IOP to develop new glaucoma therapies that target the molecular pathology in the TM.

Identifying the expression profiles of miRNAs in POAG and XFG could help us to better understand the changes in gene expression in the TM as well as gain insight into potential pathways that may be involved in the pathogenesis of the disease. The ability of miRNA manipulations to alter gene expression has raised the possibility of miRNA-based therapeutics [34,35,36,37]. Using miRNA-Seq the miRNA expression profile in the normal human trabecular meshwork has been established [38]. However, no previous studies have looked at miRNA expression changes in the TM following TGFβ treatment. Therefore, our study is the first miRNA-Seq study to evaluate the effects of TGF-β1 and -β2 treatment on miRNA expression in normal human primary TM cells. Evaluating TGFβ-responsive miRNA expression in the TM will further our understanding of the important pathways and changes involved in the pathogenesis of POAG and XFG and could lead to the development of miRNAs as new therapeutic modalities in glaucoma.

## 2. Materials and Methods

### 2.1. Human Trabecular Meshwork Culture and Characterisation

Cadaveric eyes (*n* = 4) were provided by the Liverpool Research Eye Bank, approved by the local ethics review board (RETH000833), and handled following the tenets of the Declaration of Helsinki. Donor eyes were obtained from the Royal Liverpool University Hospital mortuary. Medical history for the donor eyes was unknown, however, no donors had previous ocular surgery or a known glaucoma diagnosis. Donor eyes were excluded if the maximum post-mortem time exceeded 48 h. Primary normal human trabecular meshwork (TM) cells were isolated using the blunt dissection method as reported previously [39]. Cells were maintained in Dulbecco’s Modified Eagle Media (DMEM)-low glucose (Sigma, Gillingham, UK) supplemented with 10% foetal calf serum (Biosera, Heathfield, UK), 2 mM L-glutamine (Sigma, Gillingham, UK), Pen/Step (Sigma, Gillingham, UK), and 2.5 µg/mL Fungizone (amphotericin B, Sigma, Gillingham, UK). Samples were incubated at 37 °C (5% CO_2_ and 95% humidity). Human TM characterisation was performed as previously described [39] including dexamethasone upregulation of myocilin protein expression (polyclonal rabbit anti-myocilin primary antibody was a kind gift from Dr. W. Daniel Stamer) as previously described by our group [40].

### 2.2. TGFβ Treatment and RNA Extraction

Human TM cells between passages 4 and 6 were grown to 70–80% confluency and growth arrested overnight using serum free medium before TGF-β1 or -β2 stimulation. with either recombinant human TGF*β*1 (240-B-010, R&D Systems, Abingdon, UK) or TGFβ2 (302-B2-010, R&D Systems, UK) at a concentration of 5 ng/mL for 24 h. Total RNA was extracted from cells using the miRNeasy Mini Kit (Qiagen, Manchester, UK) following the manufacturer’s instructions. The RNA concentration was measured using the NanoDrop 2000 (Thermofisher Scientific, Horsham, UK) and RNA quality was determined by the Bioanalyser 2100 (Agilent Technologies, Stockport, UK) using an RNA 6000 Nano Kit (Agilent, Santa Clara, CA, USA).

### 2.3. Small RNA Sequencing and Data Analysis

Small RNA sequencing was performed at the Genomics Core Technology Unit (Queen’s University, Belfast, UK) using the Qiagen QIAseq miRNA Library Kit (Qiagen, Manchester, UK) to construct small RNA sequencing libraries. Sequencing was performed using a high-output sequencing kit (76 cycles) on a NextSeq500 (Illumina Inc., San Diego, CA, USA) according to the manufacturer’s instructions. The raw sequence data reads were evaluated using FastQC v0.11.9 [41] to check the quality of the reads. Cutadapt 3.0 [42] was applied to remove low-quality reads (reads with a Q score/Phred score of less than 30; 99% accuracy) and adapters to ensure only the highest-quality sequences were included for further analysis. Following quality control, the Subread and RSubread packages [43] were used to align the remaining sequences to the human genome reference (GRCh38) from the Human GENCODE Gene Set [44]. The reference database miRbase (v22.1) was used for miRNA alignment [45]. A count matrix of mapped reads for each miRNA was generated using featureCount [46]. These raw counts were used for downstream differential expression analysis. Counts data were generated for each paired treatment group and the significance of miRNA expression was compared using the generalised linear model (GLM) approach implemented by Bioconductor edgeR (v3.18), following the Trimmed Mean of M (TMM) method of normalisation [47]. miRNAs were considered to be differentially expressed if the *p*-value was less than 0.05. Volcano plots of differentially expressed miRNAs (DEmiRs) were generated using the Enhanced Volcano package in R Version 1.10.0 [41].

### 2.4. Functional and Pathway Enrichment Analysis

Following miRNA-Seq analysis, functional enrichment analysis was performed through the Database for Annotation, Visualization and Integrated Discovery (DAVID) bioinformatic package (https://david.ncifcrf.gov/; accessed on 1 June 2023) [48,49]. Firstly, to gather a list of target genes for significant miRNAs, TargetScan (https://www.targetscan.org/vert_71/; accessed on 1 June 2023) [50] and miRTarbase (https://dianalab.e-ce.uth.gr/tarbasev9; accessed on 1 June 2023) [51] were used to identify strong experimentally validated genes for significant miRNAs with a threshold of FDR < 0.05 to ensure a high confidence. The list of gene targets was added to the DAVID software (v2023q4) to assess significantly enriched pathways. Kyoto Encyclopedia of Genes and Genomes (KEGG) enrichment analysis was carried out to investigate relationships between the significantly expressed genes and their related pathways. Significance was calculated in DAVID using Fisher’s exact test to obtain *p*-values. Only pathways with a *p*-value < 0.05 were considered significant.

### 2.5. miRNA RT-qPCR Validation of miRNA-Seq Data

For miRNA analyses, 5 ng/µL of total RNA was reverse transcribed into cDNA using the miRCURY LNA RT Kit (Qiagen, UK) following manufacturer specifications. RT-qPCR was performed using miRCURY LNA SYBR Green PCR Kit (Qiagen, UK) on a LightCycler^®^480 real-time PCR system (Roche Diagnostics, Lucerne, Switzerland), following manufacturer instructions. miRNA primers were obtained from Qiagen (miRCURY LNA primer assays (Qiagen, UK) (Table S1). All miRNA expression was measured in duplicate at CT threshold levels and normalised with the average CT values of a housekeeping control; U6. Values were expressed as fold increase over the corresponding values for control by the 2-ΔΔCT method. Two independent experiments were performed, and the average (±SD) results were calculated using GraphPad Prism software (Version Prism 10.1.1) (GraphPad Software, San Diego, CA, USA). Data were expressed as the mean values ± SD and graphed using log scale. Statistical significance was analysed using a student *t*-test. Differences in the mean were considered statistically significant if *p* < 0.05.

## 3. Results

### 3.1. Descriptive Features of Small RNA-Seq Data

Three small RNA libraries (donor control, donor TGFβ1-treated and donor TGFβ2-treated) were sequenced. In total, 10+ million 75 bp reads were obtained with an average of 13.2 million reads per sample ranging from 10 to 15 million reads per sample. Only data with a Q score greater than 30 (>99.9% correct) were utilised in the mapping step of the analysis pipeline. The mapping for each sample was on average 86% and the uniformity of the mapping statistics suggests that the samples are comparable.

### 3.2. Differential Expression of TGFβ1-Responsive miRNAs in TM Cells

**Figure 1:**
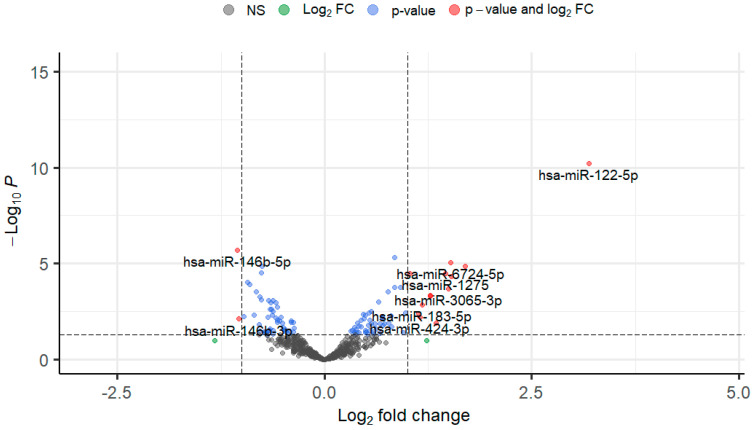
Significant differential expression of miRNAs in response to TGFβ1 treatment (5 ng/mL 24 h). Volcano plot identifying differentially expressed miRNAs in response to TGFβ1 treatment in TM cells. A threshold was applied using a statistical significance of *p* < 0.05 and log_2_FC > 1. Grey and green dots represent miRNAs failing to meet the significance cut-off. Blue dots represent miRNAs that have met the *p*-value but are below the log_2_FC criteria. Red dots represent those miRNAs that have met the threshold with a *p* < 0.05 and log_2_FC > 1. Those on the left corner of the plot are down-regulated whilst those on the right area of the plot are the up-regulated miRNAs. Grey dots = Not Significant (NS), Red dots = *p*-value < 0.05 & Log_2_FC ≥ 1, Blue dots = *p*-value < 0.05. *p*-value is represented by the dashed horizontal line and Log_2_FC is represented by the vertical dashed lines.

To determine changes in miRNA expression in the TM in response to TGFβ1, normal primary human TM cells were treated with 5 ng/mL TGFβ1 and analysed using small RNA-Seq. In total, 107 miRNAs were statistically significantly altered (*p* < 0.05), with 58 significantly up-regulated and 49 significantly down-regulated. All differentially expressed TGFβ1-induced miRNAs were graphed on a volcano plot (Figure 1) which allows for easy visual identification of miRNAs with large and statistically significant fold changes. The top 30 up-regulated and 30 down-regulated DEmiRs were ranked by fold change and are shown in Table 1 and Table 2, respectively.

### 3.3. Functional Enrichment Analysis of the TGFβ1 Differentially Expressed Genes

**Figure 2:**
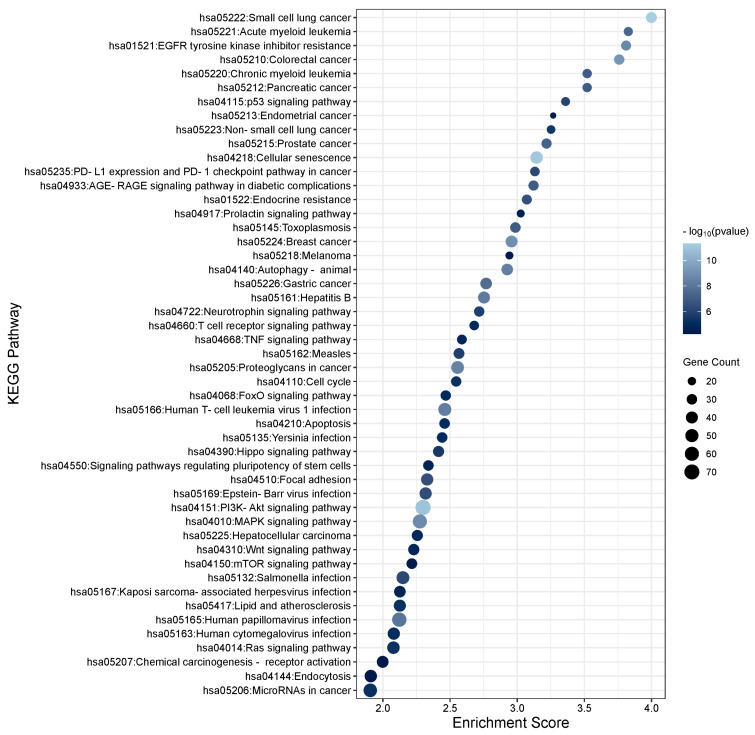
KEGG pathway analysis of significantly up-regulated TGFβ1-responsive miRNAs in TM cells. KEGG pathway analysis of the significant differentially expressed miRNAs (*p* < 0.05) was performed. The enrichment plot presents the top 30 KEGG pathway enrichment terms for differentially expressed miRNAs up-regulated in response to TGFβ1 in TM cells. The X-axis label represents Enrichment Score, the amount of differentially expressed genes enriched in the pathway, and Y-axis label represents the enriched pathway. The size and colour of the bubble represent the amount of differentially expressed genes enriched in the pathway and the enrichment significance, respectively.

**Figure 3:**
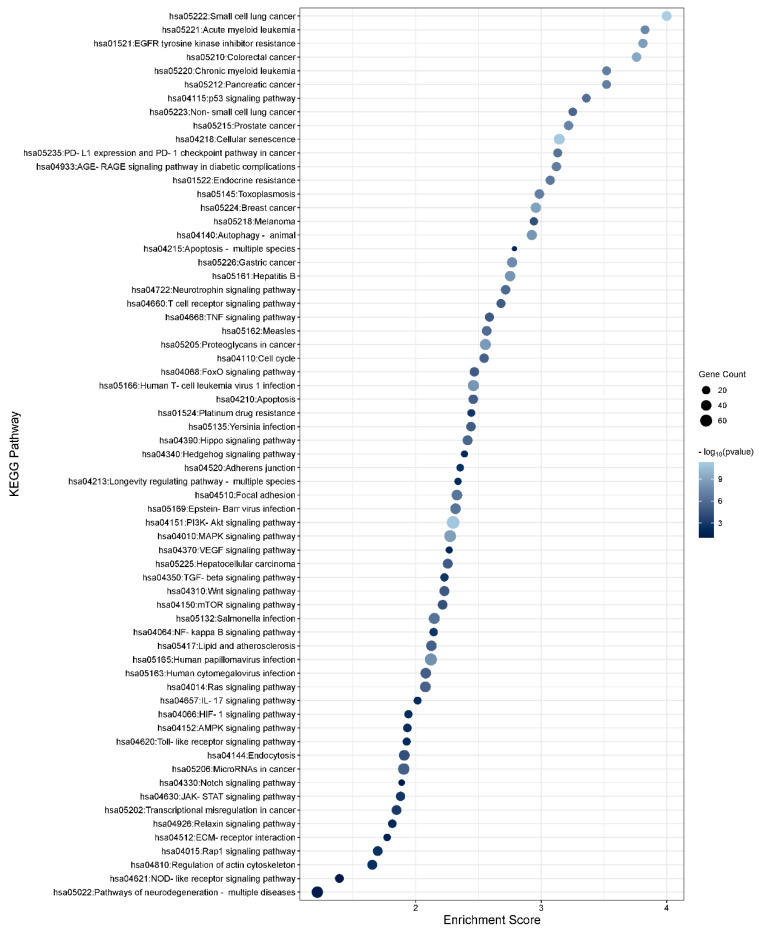
KEGG pathway analysis of significantly down-regulated TGFβ1-responsive miRNAs in TM cells. KEGG pathway analysis of the significant differentially expressed miRNAs (*p* < 0.05) was performed. The enrichment plot presents the top 30 KEGG pathway enrichment terms for differentially expressed miRNAs down-regulated in response to TGFβ1 in TM cells. The X-axis label represents Enrichment Score, the amount of differentially expressed genes enriched in the pathway, and Y-axis label represents the enriched pathway. The size and colour of the bubble represent the amount of differentially expressed genes enriched in the pathway and the enrichment significance, respectively.

To determine the function of the TGFβ1-induced DEmiRs and the potential role they play within the TM, KEGG enrichment analysis was performed using DAVID software (https://david.ncifcrf.gov; v2023q4) on the target genes of the significant DEmiRs (*p* < 0.05). To gather a list of target genes for the significant miRNAs, TargetScan and miRTarbase were used to identify strong experimentally validated genes for significant miRNAs with a threshold of FDR < 0.05 to ensure high confidence. The list of gene targets was added to the DAVID software to assess significantly enriched pathways. In total, 135 unique pathways were found to be associated with TGFβ1-regulated miRNAs. The top 30 KEGG pathways for gene targets of the up- and down-regulated TGFβ1 responsive miRNAs are shown in Figure 2 and Figure 3, respectively. Pathways relating to TGFβ signalling, MAPK signalling, PI3K-Akt signalling, Hippo signalling, Wnt signalling, focal adhesion, and regulation of the actin cytoskeleton are relevant to glaucoma/PXFG pathogenesis and are listed in Table 3 (significantly up-regulated miRNAs) and Table 4 (significantly down-regulated miRNAs) along with the associated miRNAs and gene targets. Pathways relating to TGFβ signalling, MAPK signalling, PI3K-Akt signalling, Hippo signalling, Wnt signalling, focal adhesion, and regulation of the actin cytoskeleton are relevant to glaucoma/PXFG pathogenesis and are listed in Table S2 (significantly up-regulated miRNAs) and Table S3 (significantly down-regulated miRNAs) along with the associated miRNAs and gene targets.

### 3.4. Validation of TGFβ1 Responsive DEmiRs by RT-qPCR

**Figure 4:**
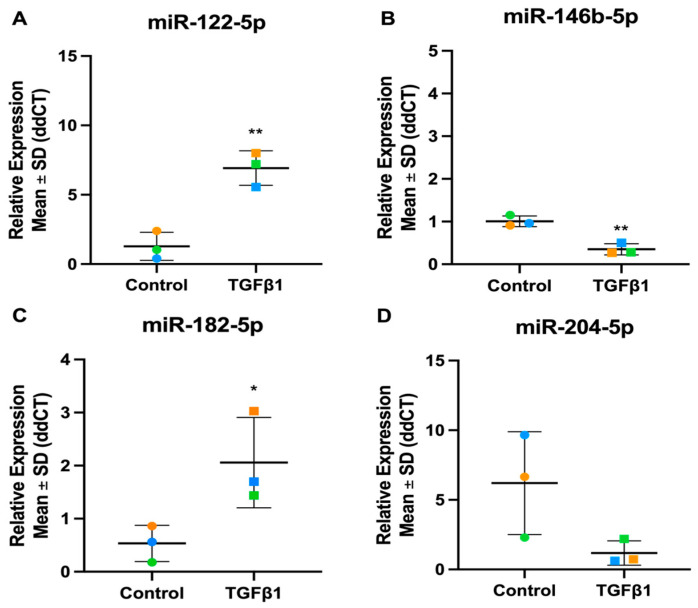
Expression levels of four candidate miRNAs in TGFβ1-stimulated TM cells. Candidate miRNAs were identified as significantly differentially expressed through miRNA-Seq. (**A**) hsa-miR-122-5p (**B**) hsa-miR-146b-5p (**C**) hsa-miR-182-5p (**D**) hsa-miR-204-5p. Vehicle controls are denoted as “Control” on graphs and TGFβ1 treated cells as “TGFβ1”. Individual values for donors 1, 2, and 3 are shown. Data were normalised to U6 control and analysed using the ΔΔCt method. Circles represent individual donor gene without TGFβ1 treatment and squares represent donors with TGFβ1 treatment. The colour of circle or square represents the individual donor human primary TM cells. An asterisk denotes significant differential gene expression after treatment (* = *p* < 0.05, ** = *p* < 0.005).

To validate the TGFβ1 miRNA-Seq results, the expression of a selected panel of significant TGFβ1 responsive miRNAs was analysed by RT-qPCR (Figure 4). The differential expression of the four miRNAs was validated by RT-qPCR: miR-122-5p (miRNA-Seq: Fold Change = 9.13, *p*-value = 6.15 × 10^−11^), miR-146b-5p (miRNA-Seq: Fold Change = 0.48, *p*-value = 1.96 × 10^−6^), miR-182-5p (miRNA-Seq: Fold Change = 1.75, *p*-value = 0.02) and miR-204-5p (miRNA-Seq: Fold Change = 0.52, *p*-value = 9.46 × 10^−5^), correlate to the expression patterns shown in the miRNA-Seq data set, confirming the reliability of the sequencing experiment.

### 3.5. Differential Expression of TGFβ2-Responsive miRNAs in TM Cells

**Figure 5:**
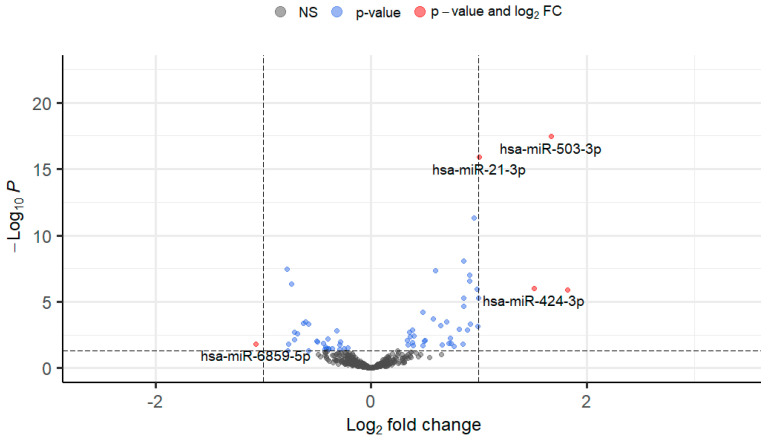
Significant differential expression of miRNAs in response to TGFβ2 treatment (5 ng/mL 24 h). Volcano Plot identifying differentially expressed miRNAs in response to TGFβ2 treatment in TM cells. A threshold was applied using a statistical significance of *p* < 0.05 and log_2_FC > 1. Grey and green dots represent miRNAs failing to meet the significance cut-off. Blue dots represent miRNAs that have met the *p*-value but are below the log_2_FC criteria. Red dots represent those miRNAs that have met the threshold with a *p* < 0.05 and log_2_FC > 1. Those on the left corner of the plot are down-regulated whilst those on the right area of the plot are the up-regulated miRNAs. Grey dots—Not Significant (NS), Red dots = *p*-value < 0.05 & Log_2_FC ≥ 1, Blue dots = *p*-value < 0.05. *p*-value is represented by the dashed horizontal line and Log_2_FC is represented by the vertical dashed lines.

To determine changes in miRNA expression in the TM in response to TGFβ2, normal primary human TM cells were treated with 5 ng/mL TGFβ2 and analysed using small RNA-Seq. In total, 67 miRNAs were statistically significantly altered (*p* < 0.05), with 38 significantly up-regulated and 28 significantly down-regulated. All differentially expressed TGFβ2-induced miRNAs were graphed on a volcano plot (Figure 5). The up-regulated and down-regulated DEmiRs were ranked by fold change and are shown in Table 3 and Table 4, respectively.

### 3.6. Functional Enrichment Analysis of the TGFβ2 Responsive Differentially Expressed Genes

**Figure 6:**
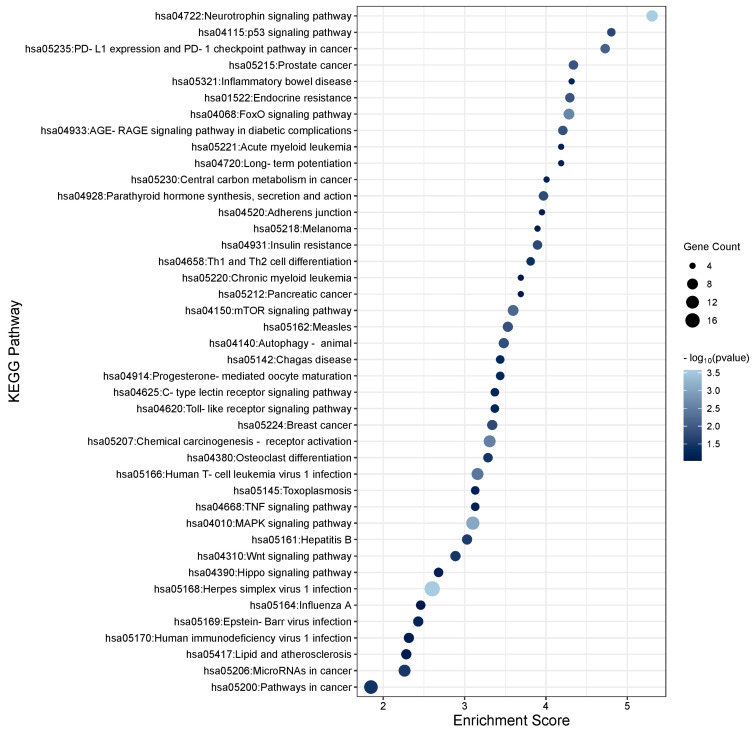
KEGG pathway analysis of significantly up-regulated TGFβ2-responsive miRNAs in TM cells. KEGG pathway analysis of the significant differentially expressed miRNAs (*p* < 0.05) was performed. The enrichment plot presents the top 30 KEGG pathway enrichment terms for differentially expressed miRNAs up-regulated in response to TGFβ2 in TM cells. The X-axis label represents Enrichment Score, the amount of differentially expressed genes enriched in the pathway, and Y-axis label represents the enriched pathway. The size and colour of the bubble represent the amount of differentially expressed genes enriched in the pathway and the enrichment significance.

**Figure 7:**
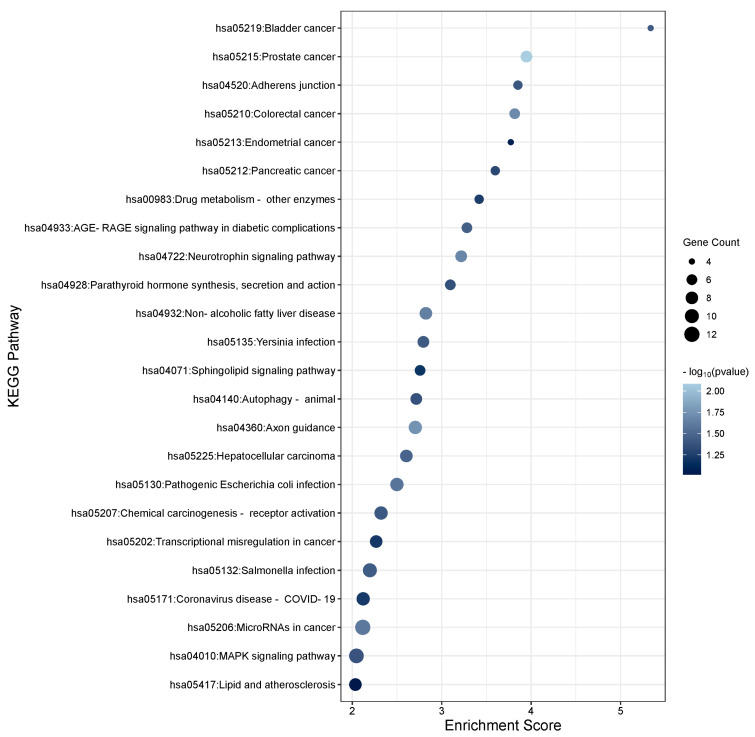
KEGG pathway analysis of significantly down-regulated TGFβ2-responsive miRNAs in TM cells. KEGG pathway analysis of the significant differentially expressed miRNAs (*p* < 0.05) was performed. The enrichment plot presents the top 30 KEGG pathway enrichment terms for differentially expressed miRNAs down-regulated in response to TGFβ2 in TM cells. The X-axis label represents Enrichment Score, the amount of differentially expressed genes enriched in the pathway, and Y-axis label represents the enriched pathway. The size and colour of the bubble represent the amount of differentially expressed genes enriched in the pathway and the enrichment significance.

To determine the function of the TGFβ2-induced DEmiRs and the potential role they play within the TM, KEGG enrichment analysis was performed using DAVID software on the target genes of the significant DEmiRs adjusting for multiple corrections (FDR < 0.05). To gather a list of target genes for the significant miRNAs, TargetScan and miRTarbase were used to identify experimentally validated genes for significant miRNAs with a threshold of FDR < 0.05 to ensure a high confidence. The genes were added to the DAVID software to assess significant pathways. In total, 58 pathways were found to be associated with TGFβ2-regulated miRNAs. The top 30 KEGG pathways for gene targets of the up- and down-regulated TGFβ2 responsive miRNAs are shown in Figure 6 and Figure 7, respectively. Pathways relating to MAPK signalling, PI3K-Akt signalling, Hippo signalling, Wnt signalling, focal adhesion, apoptosis, cellular senescence, and regulation of the actin cytoskeleton are relevant to POAG pathogenesis and are listed in Table S4 (significantly up-regulated miRNAs) and Table S5 (significantly down-regulated miRNAs) along with the associated miRNAs and gene targets.

### 3.7. Validation of TGFβ2 Responsive DEmiRs by RT-qPCR

**Figure 8:**
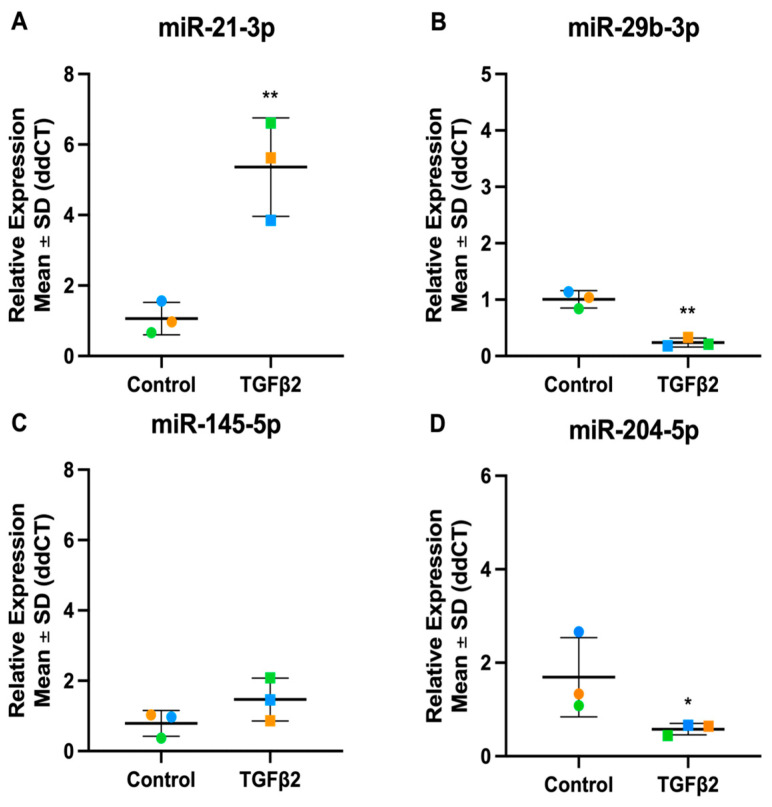
Expression levels of five candidate miRNAs in TGFβ2-stimulated TM cells. Candidate miRNAs were identified as significantly differentially expressed through miRNA-Seq. (**A**) hsa-miR-21-3p (**B**) hsa-miR-29b-3p (**C**) hsa-miR-145-5p (**D**) hsa-miR-204-5p. Vehicle controls are denoted as “Control” on graphs and TGFβ2 treated cells as “TGFβ2”. Individual values for donors 1, 2, and 3 are shown. Data were normalised to U6 control and analysed using the ΔΔCt method. Circles represent individual donor gene without TGFβ2 treatment and squares represent donors with TGFβ2 treatment. The colour of circle or square represents the individual donor human primary TM cells. An asterisk denotes significant differential gene expression after treatment (* = *p* < 0.05, ** = *p* < 0.005).

To validate the TGF*β*2 miRNA-Seq results, the expression of a selected panel of significant TGF2-responsive miRNAs was analysed by RT-qPCR (Figure 8). The differential expression of the four miRNAs was validated by RT-qPCR: miR-21-3p (miRNA-Seq: Fold Change = 2.01, *p*-value = 1.26 × 10^−16^), miR-29b-3p (miRNA-Seq: Fold Change = 0.81, *p*-value = 0.04), miR-145-5p (miRNA-Seq: Fold Change = 1.51, *p*-value = 4.34 × 10^−8^) and miR-204-5p (miRNA-Seq: Fold Change = 0.82, *p*-value = 0.01), correlate to the expression patterns shown in the miRNA-Seq dataset, again confirming the reliability of the sequencing experiment.

### 3.8. Altered miRNA Expression Grouped by miRNA Families

Several miRNAs form a miRNA family which is defined as two or more miRNAs with high sequence similarity and are derived from molecular ancestors during evolution [52]. MiRNA family members can target inter-related genes and pathways [52] and two well-studied examples in cancer and fibrosis are the miR-17-92 [53,54] and miR-29 families [55].

#### 3.8.1. miR-17-92 Family

**Figure 9:**
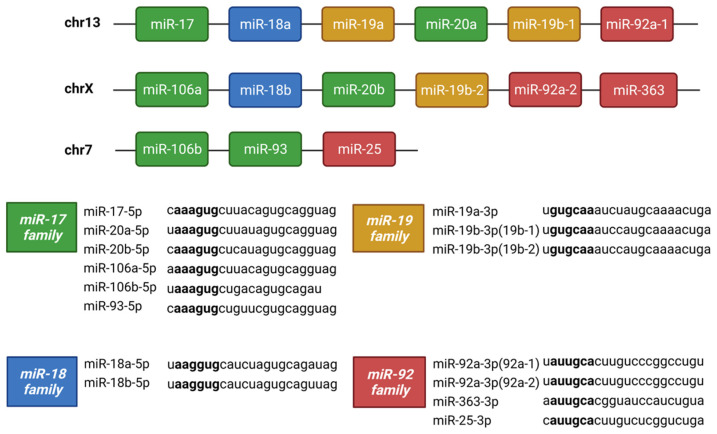
The structure of the miR-17-92 family along with its two paralogs: miR-106a-363 and miR-106b-25. The miR-17-92 cluster is located on chromosome 13 and consists of miR-17, miR-18a, miR-19a, miR-19b-1, miR-20a, and miR-92a-1. The miR-106a-363 cluster is located on chromosome X and consists of miR-106a, miR-18b, miR-20b, miR-19b-2, miR-92a-2, and miR-363. The third cluster is miR-106b-25 consisting of miR-106b, miR-93, and miR-25. These fifteen miRNAs can be grouped into four seed families: miR-17, miR-18, miR-19, and miR-92 families.

**Figure 10:**
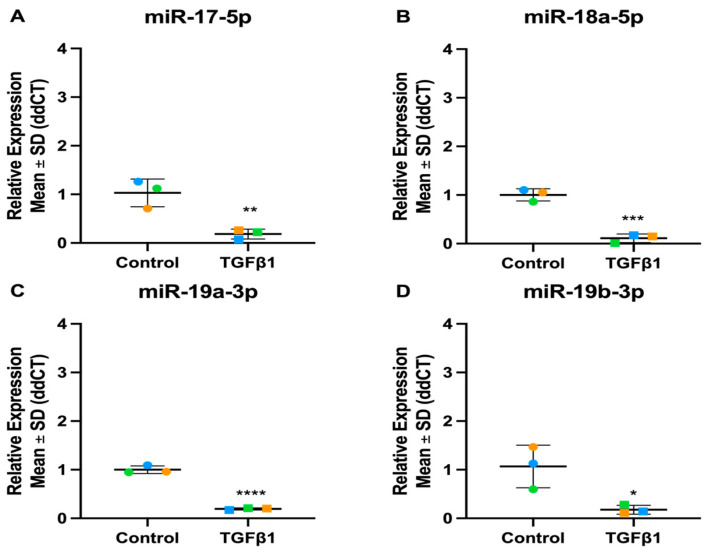

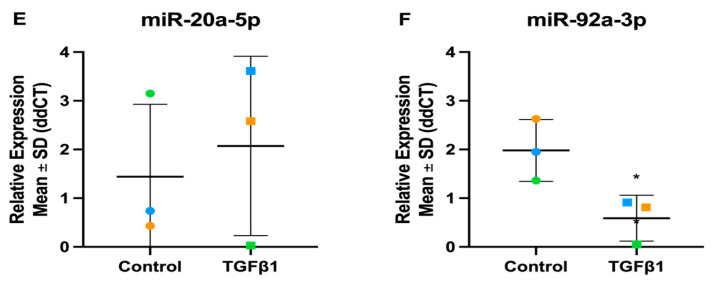
Expression levels of miR-17-92 family in TGFβ1-stimulated TM cells. (**A**) hsa-miR-17-5p (**B**) hsa-miR-18a-5p (**C**) hsa-miR-19a-3p (**D**) hsa-miR-19b-3p (**E**) hsa-miR-20a-5p (**F**) hsa-miR-92a-3p. Vehicle controls are denoted as “Control” on graphs and TGFβ1 treated cells as “TGFβ1”. Individual values for donors 1, 2, and 3 are shown. Data were normalised to U6 control and analysed using the ΔΔCt method. Circles represent individual donor gene without TGFβ1 treatment and squares represent donors with TGFβ1 treatment. The colour of circle or square represents the individual donor human primary TM cells. An asterisk denotes significant differential gene expression after treatment (* = *p* < 0.05, ** = *p* < 0.005, *** = *p* < 0.0005, **** = *p* < 0.00005).

**Figure 11:**
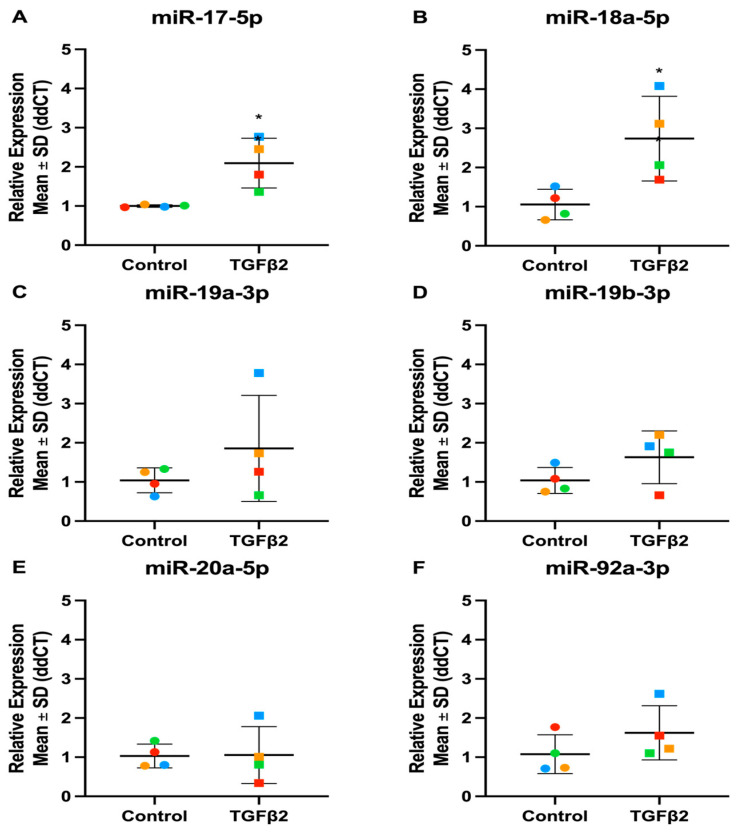
Expression levels of miR-17-92 family in TGFβ2-stimulated TM cells. (**A**) hsa-miR-17-5p (**B**) hsa-miR-18a-5p (**C**) hsa-miR-19a-3p (**D**) hsa-miR-19b-3p (**E**) hsa-miR-20a-5p (**F**) hsa-miR-92a-3p. Vehicle controls are denoted as “Control” on graphs and TGFβ2 treated cells as “TGFβ2”. Individual values for donors 1, 2, and 3 are shown. Data were normalised to U6 control and analysed using the ΔΔCt method. Circles represent individual donor gene without TGFβ2 treatment and squares represent donors with TGFβ2 treatment. The colour of circle or square represents the individual donor human primary TM cells. An asterisk denotes significant differential gene expression after treatment (* = *p* < 0.05).

The miR-17-92 family is encoded by the MIR17HG gene, which is transcribed as a polycistronic transcript that produces six mature miRNAs: miR-17, miR-18, miR-19a, miR-19b, miR-20, and miR-92 (Figure 9) [56]. Interestingly, we identified the miR-17-92 family as differentially expressed in TM cells following both TGFβ1 and -β2 stimulation. One striking difference we found between the TGFβ1 and TGFβ2 datasets was that TGFβ1 treatment appeared to have a larger effect on the family members. To confirm the differential expression of this family in TM cells, we performed RT-qPCR, treating the cells with either TGFβ1 or TGFβ2. In TGFβ1-treated TM cells, the RT-qPCR results followed the same trend as the miRNA-Seq data, apart from miR-20a-5p which showed donor variability. The RT-qPCR results in TGFβ2-treated TM cells were less significant, which followed the pattern found in the miRNA-Seq data. miR-17-5p and miR-18a-5p were both significantly up-regulated, like the miRNA-Seq data, while the rest of the family were not significantly altered (Table 5 and Figure 9, Figure 10 and Figure 11).

#### 3.8.2. miR-29 Family

**Figure 12:**
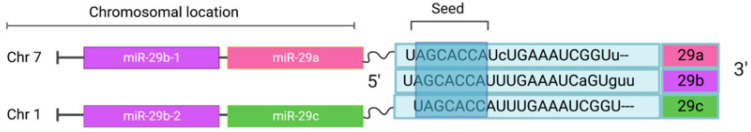
Schematic representation of the miR-29 family. Adapted from Smyth et al. [55].

**Figure 13:**
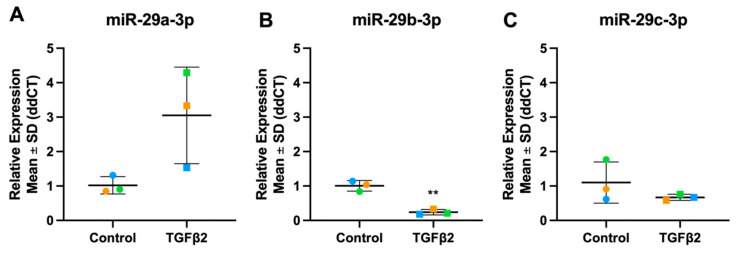
Expression levels of miR-29 family in TGFβ2-stimulated TM cells. (**A**) hsa-miR-29a-3p (**B**) hsa-miR-29b-3p (**C**) hsa-miR-29c-3p. Vehicle controls are denoted as “Control” on graphs and TGFβ2 treated cells as “TGFβ2”. Individual values for donors 1, 2, and 3 are shown. Data were normalised to U6 control and analysed using the ΔΔCt method. Circles represent individual donor gene without TGFβ2 treatment and squares represent donors with TGFβ2 treatment. The colour of circle or square represents the individual donor human primary TM cells. An asterisk denotes significant differential gene expression after treatment (** = *p* < 0.005).

The miR-29 family includes miR-29a, miR-29b-1, miR-29b-2, and miR-29c [57]. Both miR-29b-1 and miR-29b-2 have identical mature sequences and are therefore collectively referred to as miR-29b [58]. MiR-29a and -29b-1 are encoded on chromosome 7q32.3, while the miR-29b-2 and -29c cluster are found on chromosome 1q32.2 [59] (Figure 12). All four members have a common seed sequence in nucleotides 2 to 8 and largely regulate a similar group of target mRNAs. Following TGFβ2 stimulation, miR-29a was up-regulated while miR-29b and miR-29c were down-regulated, in both the miRNA-Seq dataset and RT-qPCR (Figure 13).

### 3.9. miRNA Strands

During miRNA biogenesis, one strand of the miRNA duplex is selectively loaded into the AGO protein where the specificity of the miRISC is determined based on the complementarity between the miRNA and the 3′UTR of the target mRNA [60]. The miRNA can originate from the 5′ side of the pre-miRNA to which it is referred to as the “-5p” strand or the 3′ side of the pre-miRNA, to which it is referred to as the “-3p” strand. Typically, one strand is loaded into the AGO protein becoming the functional miRNA or guide strand while the other strand is discarded. This strand is known as the miRNA* or passenger strand [61,62,63]. The fact that both strands may be loaded into the AGO protein is becoming more widely studied and either strand may form fully functioning mature miRNAs [64,65]. Therefore, it is important to know whether one or both of -5p/-3p strands of a miRNA are playing a functional role in glaucoma pathology. We were interested in understanding the effects of TGFβ on the expression of the paired -5p and -3p strands of the same miRNA in the TM cells.

**Figure 14:**
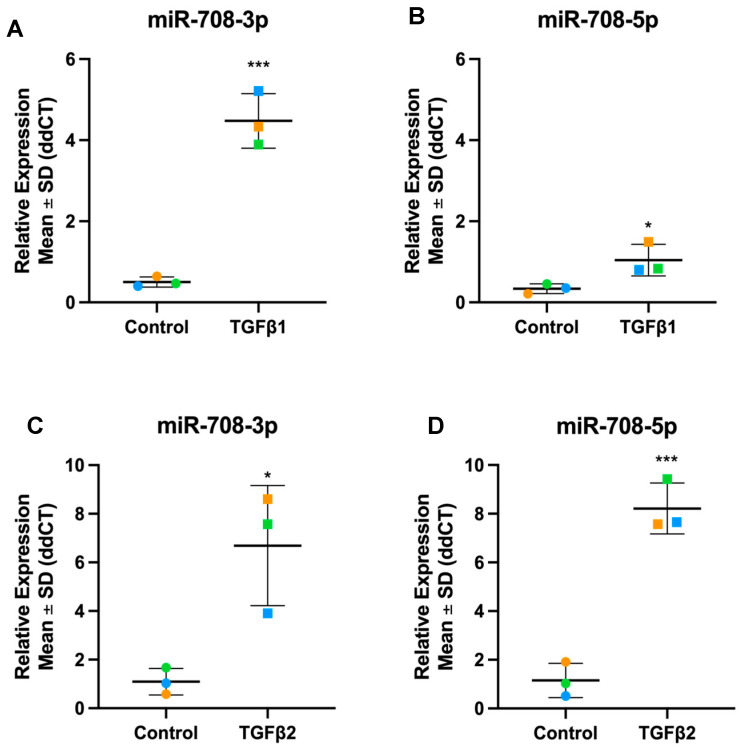
Expression levels of miR-708-5p and miR-708-3p in TGFβ1 and β2-stimulated TM cells. (**A**) hsa-miR-708-3p with TGFβ1 (**B**) hsa-miR-708-5p with TGFβ1 (**C**) hsa-miR-708-3p with TGFβ2 (**D**) hsa-miR-708-5p with TGFβ2. Individual values for donors 1, 2, and 3 are shown. Data were normalised to U6 control and analysed using the ΔΔCt method. Circles represent individual donor gene without TGFβ treatment and squares represent donors with TGFβ treatment. The colour of circle or square represents the individual donor human primary TM cells. An asterisk denotes significant differential gene expression after treatment (* = *p* < 0.05, *** = *p* < 0.0005).

**Figure 15:**
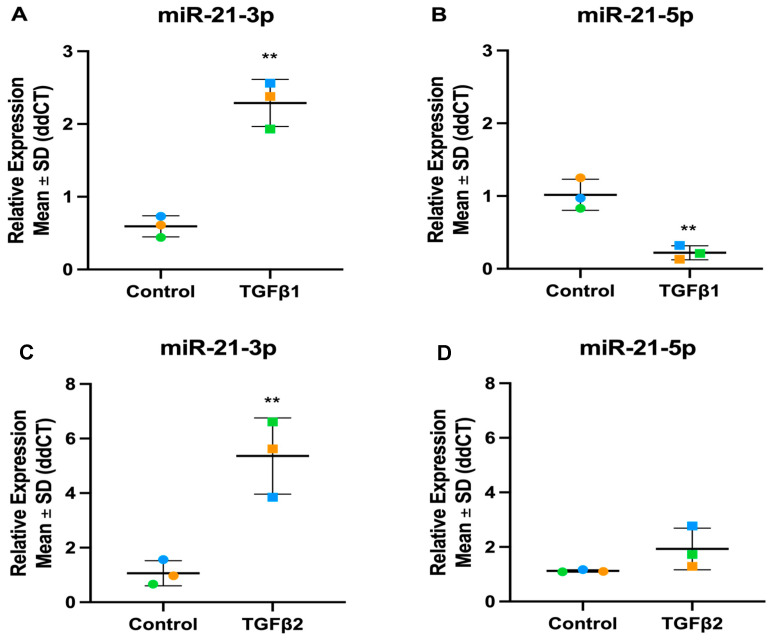
Expression levels of miR-21-3p and miR-21-5p in TGFβ1 and β2-stimulated TM cells. (**A**) hsa-miR-21-3p with TGFβ1 (**B**) hsa-miR-21-5p with TGFβ1 (**C**) has-miR-21-3p with TGFβ2 (**D**) has-miR-21-5p with TGFβ2. Individual values for donors 1, 2, and 3 are shown. Data were normalised to U6 control and analysed using the ΔΔCt method. Circles represent individual donor gene without TGFβ1 treatment and squares represent donors with TGFβ1 treatment. The colour of circle or square represents the individual donor human primary TM cells. An asterisk denotes significant differential gene expression after treatment (** = *p* < 0.005).

In our datasets, we discovered both the guide and passenger strands of several miRNAs were altered by TGFβ treatment. miR-708-3p and -5p are significantly up-regulated in TGFβ1 and TGFβ2 stimulated TM cells (Figure 14). miR-21-3p, also referred to as miR-21*, is significantly up-regulated in both TGFβ1 and TGFβ2 treated cells (Figure 15), while miR-21-5p, referred to as the miR-21 guide, was significantly down-regulated in TGFβ1 treated cells and slightly up-regulated, although not significant, in TGFβ2-treated TM cells (Figure 15).

## 4. Discussion

Understanding the role of the TGFβ-induced microRNAome in the trabecular meshwork (TM) provides insight into the molecular pathology of primary open-angle glaucoma (POAG) and pseudo-exfoliation glaucoma (XFG). Determining the specific genes and pathways impacted by dysregulated miRNA expression will support the development of miRNA-based therapeutics for glaucoma [66,67,68]. In this study, we have treated human primary TM cells with either TGFβ1 or TGFβ2, as cellular models of POAG (TGFβ2) and XFG (TGFβ1), and using small RNA sequencing, we identified 186 TGFβ1-regulated miRNAs in TM cells and 72 TGFβ2-regulated miRNAs suggesting TGFβ1 stimulation had a stronger effect on the miRNA expression profile in TM cells. Increased levels of TGFβ in the anterior segment of the eye induce fibrotic changes in the TM in glaucoma (POAG and XFG) including altered turnover of extracellular matrix (ECM) components, formation of cross-linked actin networks (CLANS), upregulation of alpha-smooth muscle actin (αSMA), aberrant formation of actin stress fibres and epithelial to mesenchymal transition (EMT) [12,17,18,69,70].

miR-122-5p was one of the highest upregulated miRNAs in response to TGFβ1 in the TM in our study. This response was also previously reported in the TM, and miR-122 was associated with the regulation of the TGFβ/Smad pathway [71]. Within the TGFβ signalling pathway, miR-122-5p has predicted target interactions with TGFβR1, TGFβR2, LTBP1, and SMAD2. The levels of miR-122-5p are significantly elevated in the aqueous humour in XFG [72] in addition to TGFβ1 [21,22,23]. miR-122 is one of the most abundant miRNAs in the liver, playing an important role in liver fibrosis [73] and other organ fibrosis targeting the TGFβ signalling pathway [74].

There was significant upregulation of miR-182-5p in TGFβ1-treated primary human TM cells. Elevated expression of miR-182-5p was reported during stress-induced premature senescence in cultured TM cells [75]. The absolute expression of miR-182-5p in the aqueous humour samples from glaucoma patients was elevated 2-fold [76]. An SNP (rs76481776) in the *MIR182* gene was associated with POAG in the NEIGHBORHOOD GWAS dataset although the mechanism linking this SNP with POAG has not been elucidated [76]. Originally described as a sensory organ-specific miRNA, miR-182 is also involved in immunity, cancer and regulation of TGFβ signalling [77,78,79]. By targeting SMAD7, a negative regulator of the TGFβ signalling pathway, miR-182-5p amplifies TGFβ induced epithelial to mesenchymal transition (EMT) and metastasis of cancer cells while inhibition of miR-182-5p reduces pulmonary fibrosis [80,81].

miR-145-5p is abundantly expressed in the TM and smooth muscle in the eye and is highly expressed in the aqueous humour in POAG patients [67,82,83]. It is a member of the miR-143/145 cluster which plays a role in the regulation of IOP [67]. miR-143/145 double knockout mice resulted in a 19% decrease in IOP [67]. miR-143/145 increases IOP by modulating actin dynamics enhancing the contractility of TM cells [67]. Manipulation of miR-143/145 levels in the TM may offer therapeutic potential in glaucoma [67].

The expression of miR-146b-5p was downregulated with TGFβ treatment. miR-146b-5p is a member of the miR-146 family of miRNAs, consisting of miR-146a-5p and miR-146b-5p. These two miRNAs only differ by two nucleotides on the 3′ end of their mature strand, sharing the same seed region [84]. During replicative senescence in human TM cells, miR-146a upregulation limited inflammatory responses [85]. miR-146a regulates the pro-inflammatory NF-κB signalling pathway by inhibiting interleukin-1 receptor-associated kinase 1 (IRAK1) to inhibit inflammation [86]. Following lentiviral intracameral delivery of miR-146a in rats, there was a sustained reduction of IOP of 4.4 ± 2.9 mmHg over 8 months [66]. The mechanism of IOP lowering was postulated as likely complex and potentially involving alterations in TGFβ signalling, ROCK inhibition, and/or NF-κB signalling [66]. While miR-146b-5p has not been studied in the TM, it also inhibited NF-κB-induced interleukin 6 (IL-6) expression in breast cancer cells [87]. This suggests that miR-146b-5p may play a similar role to miR-146a-5p in the TM.

### 4.1. miRNA Clusters and Families

The action of miRNAs can be synergistic, and this is exemplified by miRNA clusters which consist of multiple miRNAs with a common promoter resulting in co-expression and coordinated action [62,88,89]. The miR-17-92 cluster was enriched in both the TGFβ1 and TGFβ2 datasets. This cluster consists of six mature miRNAs: miR-17, miR-18a, miR-19a, miR-19b, miR-20a, and miR-92a [53]. Through gene duplication, this cluster has evolved to form two paralogs: the miR-106a-363 cluster and the miR-106b-25 cluster, shown in Figure 9 [54]. As some of the miRNAs share a seed sequence, they have been divided into four main miRNA families: the miR-17, miR-18, miR-19, and miR-92 families [54]. An important pathway targeted by members of the miR-17-92 family is the TGFβ signalling pathway [90,91]. miR-17-5p is down-regulated in response to TGFβ1 treatment in TM cells. TM cells under oxidative stress down-regulate miR-17-5p, which may regulate the proliferation and apoptosis of TM cells through its direct targeting of tumour suppressor PTEN [92], which is up-regulated in TM cells following TGFβ treatment [93]. Previous research from our group has shown miR-18a-5p expression increased in TM cells following TGFβ2, consistent with our miRNA-Seq results [94]. miR-18a-5p targets connective tissue growth factor (CTGF), which is a fibrotic gene elevated in the TM of glaucoma patients [95]. CTGF induces actin stress fibres and increases TM cell contractility by activating RhoA [96]. Lentiviral-mediated overexpression of miR-18a reduced TGFβ2-induced CTGF expression in TM cells and showed a reduction in TGFβ2-induced contraction of collagen gels [94]. miR-18a-5p is a potential miRNA therapeutic in glaucoma because of its ability to inhibit CTGF-associated increased TM cell contractility [94].

The synergistic action of miRNAs is also supported by miRNA families [89,97]. A miRNA family consists of two or more miRNAs with high sequence similarity and can be located in one or more distinct clusters [89]. The miR-29 family regulates a plethora of fibrosis-associated genes in various cell types including lungs, liver, heart, eye, and other organs [55,98]. The family is a known downstream target in the TGFβ/Smad pathway, and the phosphorylation of Smad3 by TGFβ causes miR-29 to be downregulated [55]. Our study detected down-regulation of miR-29b-3p in both TGFβ1 and TGFβ2 treated TM cells, as seen in previous reports [99,100]. Transfection of human TM cells with miR-29b-3p mimic down-regulated ECM proteins including collagens, laminin subunit gamma 1 (LAMC1), and fibrillin 1 (FBN1), and secreted protein acidic rich in cysteine (SPARC), a gene involved in ECM remodelling [101]. Alterations in SPARC, and collagens I and IV, cause significant changes in IOP in transgenic mice [102]. Our KEGG analysis identified the PI3K-Akt signalling pathway to be over-represented with miR-29b-3p expression. Overexpression of miR-29b-3p represses the PI3K-Akt pathway reducing collagen I expression in human Tenon’s ocular fibroblasts [103]. miR-29b-3p down-regulation in the TM may contribute to increased TGFβ-induced ECM components. miR-29 plays a critical role in regulating ECM production and is an anti-fibrotic miRNA [104] and miRNA-29b mimics attenuate pulmonary fibrosis in vivo [105].

### 4.2. miRNA Strands

The miRNA biogenesis pathway involves the sequential processing of the pri-miRNA into pre-miRNA and finally into a mature miRNA [106]. Following Drosha processing of pri-miRNAs in the nucleus, pre-miRNAs are exported into the cytoplasm and cleaved by Dicer, resulting in the miRNA duplex [63]. Transcription produces equal amounts of both strands of miRNA duplexes; however, their accumulation is mostly asymmetric at steady state [107]. As proposed in the oncology field [108], the results from our study highlight that both 3p- and 5p-arms from a miRNA warrant independent study.

miR-21-5p is known to be one of the most overexpressed miRNAs in response to tissue injury and to play an important role in fibrosis [109,110]. During miRNA biogenesis, pre-miR-21 is exported by Exportin 5 and processed by Dicer to release mature hsa-miR-21 (also known as hsa-miR-21-5p, the biologically dominant arm) and hsa-miR-21-3p (formerly named hsa-miR-21*), previously considered the less abundant or active miRNA strand [111,112,113,114]. Pro-fibrotic miR-21-5p binds to Smad7, an inhibitory Smad, and thus amplifies the TGFβ signalling pathway, causing fibrotic responses [115,116]. There is crosstalk between miR-21-5p and a variety of signalling pathways: TGFβ/SMAD, PI3K/AKT and ERK/MAPK signalling pathways, in the regulation of fibrotic processes [117]. A role of miR-21-5p in regulating IOP and outflow facility has been reported [118]. Topical administration of a synthetic miR-21-5p mimic increased miR-21-5p expression in the TM while reducing IOP by 17% [118]. Using RNA-sequencing and pathway analysis, with the predicted downstream target genes of miR-21-5p identified, they found a pathway involving FGF18, SMAD7, and MMP9 based on protein–protein interaction networks [118]. RT-qPCR confirmed the downregulation of SMAD7 and FGF18 by a miR-21-5p mimic suggesting that miR-21-5p targets SMAD7 and FGF18 to encourage ECM degradation by MMP9 in the TM [118]. In our data miR-21-5p expression was unaltered by TGFβ2 treatment in the TM but significantly downregulated with TGFβ1 treatment. Unexpectedly, our data show an upregulation of miR-21-3p with both TGFβ1 and -β2 treatment. There is emerging evidence for a biological role for miR-21-3p in malignancy [108,119], vascular biology [120,121] and in the regulation of TGFβ signalling [111]. In hepatocellular carcinoma, miR-21-3p regulates both TGFβ and Hippo signalling via SMAD7 and YAP1 [111]. Overexpression of miR-21-3p directly silences SMAD7 expression and reduces the stability of the SMAD7/YAP1 complex allowing YAP1 translocation to the nucleus and resultant profibrotic gene expression [111]. Further work is required to understand the role of the -5p and -3p miR-21 strands in TM pathophysiology.

miR-708 is not a widely studied miRNA in ocular tissues; however, its expression has been reported in retinal ganglion cells [122]. miR-708-5p is the more abundant strand and is involved in oncogenesis [123,124]. miR-708-5p was upregulated in the TM in response to TGFβ2 but the passenger strand (miR-708-3p) was significantly upregulated in response to TGFβ1 and -β2. A disintegrin and metalloproteinase 17 (ADAM17), which is overexpressed in fibrotic disorders [125,126,127] and is expressed in TM cells [128,129], is a direct target of miR-708-3p. By targeting ADAM17, miR-708-3p represses the GATA/STAT3 signalling pathway in idiopathic pulmonary fibrosis (IPF) reducing fibrosis [130]. GATA6 promotes fibroblast differentiation into myofibroblasts in IPF by mediating the α-SMA-inducing signal of TGFβ1 [131,132], and STAT3, is abundantly expressed in multiple fibrotic disorders [133]. In breast cancer cells, miR-708-3p inhibits EMT by directly targeting ZEB1, cadherin 2, and vimentin [134]. Therefore, miR-708-3p could present a new therapeutic target for TM fibrosis by targeting the ADAM17-GATA/STAT pathway and EMT.

### 4.3. miRNA Regulation of Signalling Pathways

A cell can simultaneously express multiple miRNAs to regulate gene expression in a holistic, intricate network with a single miRNA targeting multiple mRNAs and a single mRNA targeted by multiple miRNAs [31,62,88]. Our data can be considered in terms of specific miRNAs (miRNA-centric) but miRNAs work in networks to control cellular pathways and processes and a pathway-centric view of miRNA action is also required [135]. Several of the DEmiRs altered by TGFβ in the TM have been implicated in the regulation of the TGFβ signalling pathway [90,91,136]. Interestingly, the TGFβ signalling pathway was only significantly enriched in TGFβ1 down-regulated miRNAs in the TM. The transforming growth factor beta receptor 2 (TGFβR2) is targeted by miR-18a-5p [137], miR-20a-5p, miR-29b-3p, and miR-204-5p which were all significantly downregulated in the TM in response to TGFβ1. Smad2/3, the regulatory Smads in the canonical TGFβ signalling pathway, are also targeted by miR-18a-5p [138,139,140,141]. Lentiviral expression of miR-18a-5p in bleomycin mice presented lowered levels of phosphorylated Smad2/3 and a reduction in pulmonary fibrosis [137]. There are several overlapping miRNAs involved in the regulation of both the TGFβ signalling pathway and the Hippo signalling pathway.

The Hippo signalling pathway is considered a tumour suppressor pathway, playing important roles in cell differentiation and cell proliferation [142,143]. Our pathway enrichment analysis identified the Hippo signalling pathway as significantly enriched in both TGFβ1 and -β2 miRNA-Seq datasets in the TM, associated with both up- and down-regulated miRNAs. TGFβ2 upregulated the expression of miR-181c-5p in TM cells and miR-181c-5p inhibits Hippo signalling through its target large tumour suppressor 2 (LATS2) and Salvador Family WW Domain Containing Protein 1 (SAV1) [144,145,146]. SAV1 bind to the Macrophage-Stimulating 1/2 (MST1/2) kinases forming an enzyme complex which phosphorylates LATS1/2 [147,148]. The LATS1/2 kinases phosphorylate Yes-Associated Protein 1 (YAP), which in turn prevents nuclear translocation and signals its proteasomal degradation [149]. Therefore, miR-181c-5p disrupts the negative regulation of Hippo signalling and promotes the activation of YAP by targeting LATS2 and SAV1 [144,145]. YAP/TAZ also stimulates the nuclear accumulation of SMAD complexes to increase their transcriptional activity [150,151,152]. YAP/TAZ nuclear levels are elevated by TGFβ2 in both normal and glaucomatous human TM cells while inhibition of YAP/TAZ resulted in reduced focal adhesions, ECM remodelling and cell contractility [153]. When Hippo signalling is inhibited the nuclear translocation of YAP/TAZ induces the transcriptional activity of TEA domain (TEAD) family members, which increases the expression of CTGF which is associated with glaucoma and TM fibrosis [94,95,96].

MAPK signalling pathway and PI3K-Akt signalling pathway were two of the most enriched pathways targeted by DEmiRs in both TGFβ1 and -β2-treated TM cells. AKT1/2/3, MAPK1, PTEN, RAC1, and PIK3CG were associated with multiple DEmiRs. Rac family small GTPase 1 (RAC1), targeted by miR-146a-5p [154,155] and miR-574-3p [156], activates p38-MAPK signalling [157], increases ECM production in TM cells, and elevates SPARC and proinflammatory IL-6 in TM cells [158,159]. SPARC binds to ECM proteins and regulates the expression of matrix metalloproteinases (MMPs) [160]. SPARC expression is up-regulated by TGFβ2 in TM cells and is inhibited by the miR-29 family in the TM [100,161]. Up-regulation of IL-6 by TGFβ1 and TGFβ2 through p38-MAPK signalling affects mechanical stress in TM cells [162,163]. RAF1, targeted by miR-125a-5p [164], leads to MAP ERK kinase [165] and ERK1/2 activation [157]. ERK1/2 can elevate PAI-1 expression in the TM, ultimately leading to increased ECM production [158].

The PI3K-Akt signalling pathway can promote cell survival and suppress apoptosis [166]. As there is a decline in TM cellularity in glaucoma due to apoptosis and ECM remodelling [167,168], AKT activation may play an important role in the protection of TM cellularity. In our datasets, up-regulated miRNAs including miR-29b-1-5p [169], miR-122-5p [170,171], miR-143-3p [172], miR-182-5p [173,174], miR-214-3p [175], and miR-708-5p [176,177] are all putative targets of AKT. PTEN indirectly influences cellular proliferation and apoptosis as a major negative regulator of the Akt signalling pathway [178,179]. Interestingly, six down-regulated miRNAs in our dataset target PTEN [180,181,182,183,184,185,186,187,188,189,190]. Specifically studied in the TM, the down-regulation of miR-17-5p increases PTEN expression leading to increased apoptosis and decreased proliferation of TM cells under oxidative stress conditions [92].

Inhibition of Wnt signalling has previously been shown in glaucomatous TM cells and is associated with TM cell stiffening [191,192]. Secreted frizzled-related protein-1 (SFRP1), targeted by miR-582-3p [193] which was down-regulated in TGFβ2-treated TM cells, inhibits the Wnt signalling pathway and is associated with increased IOP [194]. Activating Wnt signalling through miRNA targets could restore the normal phenotype caused by Wnt inactivation, through repression of ECM genes (SPARC and CTGF), cross-linking genes (LOX), and inhibitors of MMPs (TIMP1 and PAI-1) [195,196].

### 4.4. miRNA-Based Therapeutics

miRNA-based therapeutics offer translational benefits compared to other treatment modalities [197,198,199,200]. Mature miRNAs are highly conserved across vertebrate species, facilitating the evaluation of identical miRNA-based therapeutics in preclinical efficacy, safety, and pharmacodynamics, and subsequent human clinical trials [198]. Intracameral delivery of miR-200c and miR-146a and topical application of miR-21-5p in rodent eyes lowers IOP and raises the possibility for the clinical translation of miRNA-based therapeutics for glaucoma [66,68,118]. Mature miRNA sequences are naturally occurring, endogenous short molecules (approximately 19–25 nucleotides) allowing several approaches to modulate miRNA levels as a therapeutic intervention [197,198]. MicroRNA mimics can restore miRNA levels and activity while mature miRNAs can be inhibited using miRNA sponges or antisense oligonucleotides (antimiRs) [197,198]. Target site masking antisense oligonucleotides can mask a specific miRNA binding site in a target mRNA/gene [197]. Chemical modification of miRNA-based therapeutics improves their pharmacokinetics and pharmacodynamics [197].

TGFβ signalling can be targeted using small molecule inhibitors of the TGF-β receptor kinases, neutralising antibodies to disrupt ligand–receptor interactions and antisense oligonucleotides (e.g., siRNA) [201,202]. TGF-β is involved in many physiological processes including cell survival, metabolism, growth, proliferation, differentiation, adhesion, migration, and death and as such is central to tissue and immune homeostasis [203]. Therefore, the critical function of TGFβ in maintaining tissue homeostasis makes targeting TGFβ a challenge and systemic inhibition of TGF-β signalling might evoke serious on-target side effects [201,203,204,205]. Gene targeting by miRNAs involves a seed region-mediated miRNA::mRNA interaction [88]. This is an important attribute that can be exploited in miRNA-based therapeutics as a single miRNA can target multiple genes with relatively weak gene suppression of 30–60% [206,207,208]. This aggregate effect on multiple genes can influence gene networks and pathways [200] making miRNA-based therapeutics superior to other approaches targeting single genes or proteins (e.g., siRNAs or small molecule inhibitors) as miRNAs can regulate complex biological processes and networks [199]. Rather than targeting a single gene or inhibiting a pathway like TGFβ with deleterious effects, miRNA-based therapeutics can fine-tune signalling pathways dampening responses [198,199,200]. In our data, we have shown the enriched pathways and the key miRNAs and their mRNA targets in TM cells stimulated with either TGF-β1 or -β2. This is important for determining which gene or pathway to target and with which miRNA or miRNAs because miRNA action is cell and context-specific. These data are also important for determining on-target specificity and undesired off-target and on-target effects [197,200]. Future studies assessing the miRNA::mRNA interactome using in silico and in vitro approaches can help characterise the “miRNA targetome” to support the development of miRNA-based therapeutics [200].

The delivery of miRNA therapeutics to the TM also poses challenges including penetration of the corneal barrier, prevention of premature degradation, and specific targeting of TM cells. A solution to these issues already exists in the use of nanoparticle delivery systems [209,210]. Nanoparticles and nanocarriers can increase the stability of the miRNA molecule before being absorbed by the cell, essentially acting like a vesicle. Furthermore, nanoparticles can be coated to allow more specific targeting of a particular tissue [209]. A TM-targeting coating for nanoparticles has already been developed using hyaluronan, which has been used to deliver a CTGF-targeting siRNA to the TM of ex vivo mouse, porcine, and human eyes [209]. This specific targeting of the TM would also help prevent off-target effects in the eye by using cell-specific miRNA modulation [197].

## 5. Conclusions

To our knowledge, this is the first study reporting a genome-wide miRNA expression in primary normal human TM cell samples following either TGFβ1 or -β2 stimulation using miRNA-Seq. We have identified differentially expressed miRNAs that could target genes associated with the pathogenesis of POAG and XFG, and the identified enriched pathways associated with these miRNAs. Access to diseased TM disease from glaucoma patients is limited but would add to our understanding of miRNA dysregulation in the TM and disease pathogenesis. Further work to identify the miRNA::mRNA interactome is required to fully understand the TGFβ induced microRNAome of the TM. This knowledge is also required to develop miRNA-based therapeutics for POAG and XFG which will require consideration of miRNA-centric and pathway-centric effects. There are several challenges to overcome to develop miRNA therapeutics to reduce IOP in glaucoma [41], but early pre-clinical studies are encouraging [66,68,118,210].

## Figures and Tables

**Table 1:** Top 30 significantly up-regulated miRNAs in response to TGFβ1 (5 ng/mL) treatment for 24 h. MiRNAs are ranked by Fold Change. Significance defined as *p*-value < 0.05. log_2_FC = Log_2_ Fold Change.

miRNAs	Fold Change	Log_2_FC	*p*-Value
hsa-miR-122-5p	9.13	3.19	6.15 × 10^−11^
hsa-miR-139-5p	3.25	1.70	1.38 × 10^−5^
hsa-miR-3065-5p	2.89	1.53	4.57 × 10^−5^
hsa-miR-6724-5p	2.87	1.52	8.51 × 10^−6^
hsa-miR-3065-3p	2.82	1.49	0.0002
hsa-miR-1275	2.75	1.46	3.19 × 10^−5^
hsa-miR-122b-5p	2.53	1.34	0.013
hsa-miR-10395-5p	2.42	1.28	0.0005
hsa-miR-10395-3p	2.42	1.28	0.0005
hsa-miR-183-5p	2.26	1.17	0.001
hsa-miR-424-3p	2.22	1.15	0.006
hsa-miR-10401-5p	2.19	1.13	0.004
hsa-miR-23a-5p	2.04	1.03	3.24 × 10^−5^
hsa-miR-6716-3p	1.97	0.98	0.004
hsa-miR-503-3p	1.93	0.95	0.04
hsa-miR-146a-5p	1.89	0.92	0.0002
hsa-miR-181b-5p	1.80	0.85	4.79 × 10^−6^
hsa-miR-708-5p	1.79	0.84	0.0002
hsa-miR-182-5p	1.75	0.81	0.02
hsa-miR-543	1.74	0.80	0.005
hsa-miR-3679-5p	1.71	0.77	0.01
hsa-miR-210-3p	1.70	0.77	0.0003
hsa-miR-216a-5p	1.64	0.72	0.01
hsa-miR-365a-5p	1.63	0.71	0.01
hsa-miR-129-5p	1.62	0.69	0.006
hsa-miR-10401-3p	1.62	0.69	0.007
hsa-miR-10527-5p	1.59	0.67	0.01
hsa-miR-181b-3p	1.59	0.67	0.04
hsa-miR-24-2-5p	1.57	0.65	0.001
hsa-miR-495-3p	1.55	0.63	0.02

**Table 2:** Top 30 significantly down-regulated miRNAs in response to TGFβ1 (5 ng/mL) treatment for 24 h. MiRNAs are ranked by Fold Change. Significance defined as *p*-value < 0.05. log_2_FC = Log_2_ Fold Change.

miRNAs	Fold Change	Log_2_FC	*p*-Value
hsa-miR-146b-5p	0.48	−1.05	1.96 × 10^−6^
hsa-miR-146b-3p	0.49	−1.03	0.008
hsa-miR-651-5p	0.51	−0.98	0.005
hsa-miR-204-5p	0.52	−0.93	9.46 × 10^−5^
hsa-miR-99a-5p	0.53	−0.91	0.0001
hsa-miR-218-1-3p	0.56	−0.85	0.005
hsa-miR-660-5p	0.56	−0.83	0.0003
hsa-miR-549a-3p	0.58	−0.79	0.02
hsa-miR-26a-1-3p	0.58	−0.78	0.0005
hsa-miR-500b-3p	0.58	−0.78	0.03
hsa-miR-15a-5p	0.59	−0.76	2.90 × 10^−5^
hsa-miR-452-3p	0.59	−0.76	0.0008
hsa-miR-218-5p	0.59	−0.75	1.37 × 10^−5^
hsa-miR-26a-2-3p	0.60	−0.73	0.035
hsa-miR-1255a	0.61	−0.71	0.036
hsa-miR-502-5p	0.62	−0.70	0.04
hsa-miR-99a-3p	0.62	−0.69	0.03
hsa-miR-20b-5p	0.62	−0.6	0.05
hsa-miR-9985	0.62	−0.69	0.006
hsa-miR-195-5p	0.63	−0.68	0.0008
hsa-let-7c-3p	0.63	−0.67	0.03
hsa-miR-19a-3p	0.63	−0.66	0.002
hsa-miR-335-3p	0.63	−0.66	0.025
hsa-miR-19b-3p	0.64	−0.65	0.001
hsa-miR-335-5p	0.64	−0.64	0.002
hsa-miR-29b-3p	0.65	−0.63	0.005
hsa-miR-497-5p	0.65	−0.62	0.003
hsa-miR-20a-3p	0.65	−0.62	0.04
hsa-miR-454-5p	0.65	−0.62	0.03
hsa-miR-101-3p	0.65	−0.62	0.0009

**Table 3:** Top 30 significantly up-regulated miRNAs in response to TGFβ2 (5 ng/mL) treatment for 24 h. MiRNAs are ranked by Fold Change. Significance defined as *p*-value < 0.05. log_2_FC = Log_2_ Fold Change.

miRNA Name	Fold Change	Log_2_FC	*p*-Value
hsa-miR-181b-2-3p	3.54	1.82	1.27 × 10^−6^
hsa-miR-503-3p	3.18	1.67	3.60 × 10^−18^
hsa-miR-424-3p	2.86	1.52	1.05 × 10^−6^
hsa-miR-21-3p	2.01	1.01	1.26 × 10^−16^
hsa-miR-708-3p	1.99	0.99	5.17 × 10^−6^
hsa-miR-6716-3p	1.99	0.99	0.0007
hsa-miR-503-5p	1.98	0.99	1.18 × 10^−6^
hsa-miR-27a-5p	1.904	0.95	4.55 × 10^−12^
hsa-miR-6724-5p	1.90	0.92	0.0005
hsa-miR-145-3p	1.89	0.92	2.71 × 10^−7^
hsa-miR-181a-2-3p	1.89	0.92	9.8 × 10^−8^
hsa-miR-3187-3p	1.86	0.89	0.001
hsa-miR-24-2-5p	1.82	0.86	5.526 × 10^−6^
hsa-miR-143-5p	1.82	0.86	8.45 × 10^−9^
hsa-miR-708-5p	1.81	0.86	2.20 × 10^−5^
hsa-miR-3179	1.81	0.85	0.015
hsa-miR-3065-3p	1.77	0.82	0.001
hsa-miR-10401-5p	1.71	0.77	0.02
hsa-miR-5701	1.67	0.74	0.01
hsa-miR-216a-5p	1.67	0.74	0.006
hsa-miR-135a-5p	1.65	0.72	0.01
hsa-miR-214-5p	1.63	0.70	0.0003
hsa-miR-23a-5p	1.58	0.66	0.02
hsa-miR-3065-5p	1.579	0.65	0.00064297
hsa-miR-145-5p	1.51	0.60	4.34 × 10^−8^
hsa-miR-181b-5p	1.49	0.58	0.0002
hsa-miR-199b-5p	1.41	0.50	0.008
hsa-miR-424-5p	1.41	0.50	0.009
hsa-miR-210-3p	1.40	0.48	6.48 × 10^−5^
hsa-miR-6511a-3p	1.39	0.48	0.02

**Table 4:** Top 30 significantly down-regulated miRNAs in response to TGFβ2 (5 ng/mL) treatment for 24 h. MiRNAs are ranked by Fold Change. Significance defined as *p*-value < 0.05. log_2_FC = Log_2_ Fold Change.

miRNA Name	Fold Change	Log_2_FC	*p*-Value
hsa-miR-6859-5p	0.48	−1.07	0.014
hsa-miR-218-1-3p	0.58	−0.78	3.56 × 10^−8^
hsa-miR-20b-5p	0.59	−0.77	0.049
hsa-miR-15a-3p	0.59	−0.77	0.015
hsa-miR-26a-1-3p	0.60	−0.74	4.51 × 10^−7^
hsa-miR-744-3p	0.61	−0.71	0.002
hsa-miR-760	0.61	−0.71	0.007
hsa-miR-3613-5p	0.62	−0.69	0.002
hsa-miR-485-5p	0.65	−0.62	0.0004
hsa-miR-3618	0.66	−0.61	0.0003
hsa-miR-96-5p	0.67	−0.58	0.05
hsa-let-7c-3p	0.67	−0.58	0.0005
hsa-miR-26a-2-3p	0.71	−0.50	0.01
hsa-miR-454-5p	0.71	−0.50	0.01
hsa-miR-452-3p	0.74	−0.44	0.01
hsa-miR-302b-3p	0.74	−0.43	0.04
hsa-miR-379-3p	0.75	−0.42	0.02
hsa-miR-200a-3p	0.75	−0.41	0.03
hsa-miR-330-3p	0.76	−0.40	0.006
hsa-miR-652-3p	0.76	−0.39	0.04
hsa-miR-330-5p	0.76	−0.39	0.036
hsa-miR-582-3p	0.78	−0.3596112	0.035
hsa-miR-218-5p	0.80	−0.32	0.0015
hsa-miR-324-3p	0.82	−0.29	0.02
hsa-miR-29b-3p	0.82	−0.29	0.04
hsa-miR-204-5p	0.82	−0.28	0.01
hsa-miR-887-3p	0.84	−0.25	0.03
hsa-miR-138-5p	0.86	−0.21	0.03

**Table 5:** Fold Change and *p*-value of the miR-17-92 family members in the miRNA-Seq datasets.

miRNA	TGFβ1 Dataset		TGFβ2 Dataset	
	Fold Change	*p*-Value	Fold Change	*p*-Value
hsa-miR-17-5p	0.77	0.060	1.07	0.510
hsa-miR-18a-5p	0.67	0.007	1.13	0.222
hsa-miR-19a-3p	0.63	0.002	0.89	0.360
hsa-miR-19b-3p	0.64	0.001	0.96	0.765
hsa-miR-20a-5p	0.67	0.002	0.91	0.255
hsa-miR-92a-3p	0.83	0.160	0.92	0.361

## Data Availability

Data are contained within the article. Raw RNA-Seq data were deposited and released in the SRA database: https://www.ncbi.nlm.nih.gov/sra/PRJNA1099874 (accessed on 8 June 2024).

## Supplementary Materials

The following supporting information can be downloaded at: https://www.mdpi.com/article/10.3390/cells13121060/s1, Table S1. RT-qPCR miRCURY LNA miRNA primer assays sequences. Table S2. Significantly up-regulated TGFβ1-responsive miRNAs associated with glaucoma-related signalling pathways and their gene targets. Table S3. Significantly down-regulated TGFβ1-responsive miRNAs associated with glaucoma-related signalling pathways and their gene targets. Table S4. Significantly up-regulated TGFβ2-reponsive miRNAs associated with glaucoma-related signalling pathways and their gene targets. Table S5. Significantly down-regulated TGFβ2-responsive miRNAs associated with glaucoma-related signalling pathways and their gene targets. Table S6. Significantly down-regulated TGFβ2-responsive miRNAs associated with glaucoma-related signalling pathways and their gene targets.
