# Supplementary material for: The TGFβ Induced MicroRNAome of the Trabecular Meshwork
Source: Cells. 2024 Jun 19;13(12):1060. doi: 10.3390/cells13121060 (PMC11201560; doi:10.3390/cells13121060)
Supplement: Supplementary file 1 [file cells-13-01060-s001.zip › cells-3016372-supplementary.pdf]

Table S1. RT-qPCR miRCURY LNA miRNA primer assays sequences.

miRNA Name	Mature miRNA Sequence
**hsa-miR-17-5p (MIMAT0000070)**	5'CAAAGUGCUUACAGUGCAGGUAG
**hsa-miR-18a-5p (MIMAT0000072)**	5'UAAGGUGCAUCUAGUGCAGAUAG
**hsa-miR-19a-3p (MIMAT0000073)**	5'UGUGCAAAUCUAUGCAAAACUGA
**hsa-miR-19b-3p (MIMAT0000074)**	5'UGUGCAAAUCCAUGCAAAACUGA
**hsa-miR-20a-5p (MIMAT0000075)**	5'UAAAGUGCUUAUAGUGCAGGUAG
**hsa-miR-21-3p (MIMAT0004494)**	5'CAACACCAGUCGAUGGGCUGU
**hsa-miR-21-5p (MIMAT0000076)**	5'UAGCUUAUCAGACUGAUGUUGA
**hsa-miR-29a-3p** (MIMAT0000086)	5'UAGCACCAUCUGAAAUCGGUUA
**hsa-miR-29b-3p (MIMAT0000100)**	5'UAGCACCAUUUGAAAUCAGUGUU
**hsa-miR-29c-3p (MIMAT0000681)**	5'UAGCACCAUUUGAAAUCGGUUA
**hsa-miR-92a-3p (MIMAT0000092)**	5'UAUUGCACUUGUCCCGGCCUGU
**hsa-miR-122-5p (MIMAT0000421)**	5'UGGAGUGUGACAAUGGUGUUUG
**hsa-miR-145-5p (MIMAT0000437)**	5'GUCCAGUUUUCCCAGGAAUCCCU
**hsa-miR-146b-5p (MIMAT0002809)**	5'UGAGAACUGAAUUCCAUAGGCUG
**hsa-miR-182-5p (MIMAT0000259)**	5'UUUGGCAAUGGUAGAACUCACACU
**hsa-miR-204-5p (MIMAT0000265)**	5'UUCCCUUUGUCAUCCUAUGCCU
**hsa-miR-708-3p (MIMAT0004927)**	5'CAACUAGACUGUGAGCUUCUAG
**hsa-miR-708-5p (MIMAT0004926)**	5'AAGGAGCUUACAAUCUAGCUGGG

Table S2. Significantly up-regulated TGFβ1-responsive miRNAs associated with glaucoma-related signalling pathways and their gene targets.

KEGG Pathway	miRNA	Gene Targets
**HIF-1 signalling pathway**	hsa-miR-99b-5p	IGF1R, MTOR
	hsa-miR-122-5p	AKT1, IGF1R, MTOR, PIK3CG, VHL
	hsa-miR-125a-5p	AKT1, BCL2, CDKN1A, EDN1, EGFR, EIF4EBP1, ERBB2, HK2, IFNG, MTOR, PIK3CG, STAT3, VEGFA
	hsa-miR-139-5p	BCL2, IGF1R, NFKB1, PIK3CA
	hsa-miR-181a-5p	BCL2, CDKN1A, CDKN1B, IFNG, MAP2K1, MAPK1, STAT3, TIMP1

	hsa-miR-182-5p	AKT1, BCL2, CDKN1B, IGF1R, LDHA, NFKB1, STAT3, VHL
	hsa-miR-193a-5p	ERBB2, MTOR, PIK3R3
	hsa-miR-503-5p	BCL2, CDKN1A, IGF1R, PIK3R1, VEGFA
	hsa-miR-574-3p	CUL2, EGFR, EP300
	hsa-miR-708-5p	AKT1, AKT2, BCL2
**AGE-RAGE signalling pathway in diabetic complications**	hsa-miR-125a-5p	AKT1, EGFR, JAK2, MAPK14, RAF1, SMAD2, STAT3
	hsa-miR-146a-5p	EGFR, NFKB1, RAC1, RHOA, ROCK1, SMAD2, STAT1
	hsa-miR-216a-5p	CDC42, JAK2
	hsa-miR-543	MMP7, NOS3
	hsa-miR-574-3p	EGFR, RAC1
	hsa-miR-708-5p	AKT1, MMP2, SMAD3
**Hippo signalling pathway**	hsa-miR-23b-5p	SMAD3, TGFB2, TGFBR2
	hsa-miR-182-5p	APC, BMPR2, CCND1, CSNK1E, CTNNA1, SMAD4, SMAD7, YWHAG
	hsa-miR-183-5p	BTRC, GSK3B, PPP2CB, SMAD4, SNAI2
	hsa-miR-574-3p	SMAD4, TGFB1
	hsa-miR-708-5p	BIRC5, CCND1, SMAD3
	hsa-miR-744-5p	GSK3B, MYC, NKD1
**Neurotrophin signalling pathway**	hsa-miR-122-5p	AKT1, BAX, PIK3CG
	hsa-miR-125a-5p	AKT1, BCL2, MAPK14, NTRK3, PIK3CG, RAF1, TP53, TRAF6
	hsa-miR-139-5p	BCL2, HRAS, JUN, NFKB1, PIK3CA, RAP1B
	hsa-miR-146a-5p	IRAK1, IRAK2, NFKB1, RAC1, RHOA, SOS1, TRAF6
	hsa-miR-181a-5p	BAX, BCL2, HRAS, KRAS, MAP2K1, MAPK1, NRAS, PRKCD, PTPN11, RAP1B
	hsa-miR-182-5p	AKT1, BCL2, FOXO3, FRS2, IRAK1, IRS1, MAPK8, NFKB1, SHC1
	hsa-miR-708-5p	AKT1, AKT2, BCL2
**PI3K-Akt signalling pathway**	hsa-miR-99b-5p	IGF1R, MTOR
	hsa-miR-122-5p	AKT1, CDK4, CREB1, G6PC3, GYS1, IGF1R, MTOR, MYC, PIK3CG
	hsa-miR-125a-5p	AKT1, BCL2, CDKN1A, EGFR, EIF4EBP1, JAK2, MCL1, MTOR, PIK3CG, RAF1, TP53, VEGFA
	hsa-miR-139-5p	BCL2, HRAS, IGF1R, MCL1, MET, NFKB1, PIK3CA

	hsa-miR-146a-5p	BRCA1, CCND1, CCND2, EGFR, IL6, LAMC2, NFKB1, RAC1, SOS1, TLR2, TLR4
	hsa-miR-181a-5p	BCL2, BCL2L11, CDKN1A, CDKN1B, DDIT4, HRAS, KRAS, MAP2K1, MAPK1, MCL1, NRAS, PHLPP2, PTEN
	hsa-miR-181b-5p	BCL2, BCL2L11, CREB1, IGF1R, MAP2K1, MCL1, PTEN, SPP1, TCL1A
	hsa-miR-182-5p	BCL2, CCND2, CDKN1B, CREB1, CREB5, FGF9, FOXO3, GSK3B, PTEN, THBS1
	hsa-miR-503-5p	BCL2, CCND1, CCND3, CCNE1, CCNE2, CDKN1A, FGF2, FGF8, FGFR1, IGF1R, IKBKB, MYB, PIK3R1, VEGFA
	hsa-miR-543	KRAS, NOS3, PTK2
	hsa-miR-574-3p	EGFR, RAC1, RXRA
	hsa-miR-708-5p	AKT1, AKT2, BCL2, CCND1, IKBKG
**AMPK signalling pathway**	hsa-miR-122-5p	AKT1, CREB1, G6PC3, GYS1, IGF1R, MTOR, PIK3CG
	hsa-miR-125a-5p	AKT1, CDKN1A, EIF4EBP1, ELAVL1, MTOR, PIK3CG, TP53
	hsa-miR-182-5p	AKT1, CCND1, CREB5, FOXO1, FOXO3, IGF1R, IRS4, SCD
	hsa-miR-193a-5p	MTOR, PIK3R3
	hsa-miR-708-5p	AKT1, AKT2
**JAK-STAT signalling pathway**	hsa-miR-125a-5p	AKT1, IFNG, JAK2, LIFR, PIK3CG, STAT3
	hsa-miR-216a-5p	CBL, JAK2
	hsa-miR-708-5p	AKT1, AKT2, CCND1, CNTFR
**Cell cycle**	hsa-miR-122-5p	CDK4, E2F1, MYC, ORC2, TFDP2
	hsa-miR-182-5p	CCND1, CDK4, CHEK2, E2F1, MCM3, RB1, SMAD4, YWHAG
	hsa-miR-503-5p	CCND1, CCND3, CCNE1, CCNE2, CDC14A, CDC25A, CDKN1A, CHEK1, E2F3, WEE1
	hsa-miR-708-5p	AKT1, AKT2, BIRC5, CCND1
**FoxO signalling pathway**	hsa-miR-21-3p	FASLG, PTEN, STAT3
	hsa-miR-23b-5p	SMAD3, TGFB2, TGFBR2
	hsa-miR-122-5p	AKT1, G6PC3, IGF1R, PIK3CG
	hsa-miR-125a-5p	AKT1, CDKN1A, EGFR, MAPK14, PIK3CG, RAF1, SMAD2, SMAD4, STAT3
	hsa-miR-146a-5p	CCND1, CCND2, EGFR, IL6, SMAD2, SMAD4, SOS1, TGFB1
	hsa-miR-181a-5p	ATM, BCL2L11, CDKN1A, CDKN1B, HRAS, KRAS, MAP2K1, MAPK1, NLK, NRAS, PTEN, SIRT1, STAT3, TGFBR1

	hsa-miR-181b-5p	ATM, BCL2L11, IGF1R, MAP2K1, NLK, PTEN, SIRT1
	hsa-miR-182-5p	AKT1, CCND1, CDKN1B, FOXO1, FOXO3, IGF1R, IRS1, MAPK8, SMAD4
	hsa-miR-503-5p	CCND1, CDKN1A, IGF1R, IKBKB, PIK3R1
	hsa-miR-543	KRAS, SIRT1
	hsa-miR-574-3p	EGFR, EP300, SMAD4, TGFB1
	hsa-miR-708-5p	AKT1, AKT2, CCND1, SMAD3
**NF-kappa B signalling pathway**	hsa-miR-23b-5p	CHUK, PLAU, TAB2, TAB3
	hsa-miR-146a-5p	CD40LG, CXCL12, CXCL8, ICAM1, IRAK1, NFKB1, PTGS2, TLR4, TRAF6
	hsa-miR-503-5p	BCL2, CD40, IKBKB, TNFRSF11A
	hsa-miR-708-5p	BCL2, IKBKG, PARP1
**Apoptosis**	hsa-miR-122-5p	AKT1, BAX, CASP7, PIK3CG
	hsa-miR-139-5p	BCL2, NFKB1, PIK3CA
	hsa-miR-146a-5p	CASP7, FADD, FAS, IRAK1, IRAK2, NFKB1
	hsa-miR-181a-5p	ATM, BAX, BCL2, PPP3CA, XIAP
	hsa-miR-182-5p	AKT1, BCL2, NFKB1, PPP3R1, PRKACB
	hsa-miR-503-5p	BCL2, IKBKB, PIK3R1
	hsa-miR-708-5p	AKT1, AKT2, BCL2, IKBKG
	hsa-miR-23b-5p	TGFB2, TGFBR2
**MAPK signalling pathway**	hsa-miR-139-5p	FOS, HRAS, JUN, NFKB1, RAP1B
	hsa-miR-146a-5p	DUSP1, EGFR, FAS, NFKB1, RAC1, SOS1, TGFB1, TRAF6
	hsa-miR-181a-5p	DUSP5, DUSP6, FOS, HRAS, KRAS, MAP2K1, MAPK1, NLK, NRAS, PPP3CA, RAP1B, TGFBR1
	hsa-miR-574-3p	EGFR, RAC1, TGFB1
	hsa-miR-125a-5p	AKT1, BCL2, EGFR, ERBB2, PIK3CG, RAF1, VEGFA
**Focal adhesion**	hsa-miR-122-5p	AKT1, IGF1R, PAK1, PIK3CG
	hsa-miR-139-5p	BCL2, HRAS, IGF1R, JUN, MET, PIK3CA, RAP1B, ROCK2
	hsa-miR-146a-5p	CCND1, CCND2, EGFR, LAMC2, RAC1, RHOA, ROCK1, SOS1
	hsa-miR-181a-5p	BCL2, CTNNB1, HRAS, MAP2K1, MAPK1, PTEN, RAP1B, XIAP
	hsa-miR-181b-5p	BCL2, IGF1R, MAP2K1, PTEN, RAP1B, SPP1, XIAP

	hsa-miR-182-5p	AKT1, BCL2, CCND1, IGF1R, ITGB1, LAMC1, MAPK8, MET, ZYX
	hsa-miR-574-3p	EGFR, RAC1
	hsa-miR-708-5p	AKT1, AKT2, BCL2, CCND1
	hsa-miR-744-5p	ARHGAP5, GSK3B
**mTOR signalling pathway**	hsa-miR-122-5p	AKT1, MTOR, PIK3CG
	hsa-miR-125a-5p	AKT1, EIF4EBP1, MTOR, PIK3CG, VEGFA
	hsa-miR-129-5p	IGF1, MAPK1, PDPK1
	hsa-miR-139-5p	FOS, JUN, NFKB1, PIK3CA
	hsa-miR-182-5p	AKT1, EIF4B, IRS1, RRAGA
	hsa-miR-193a-5p	MTOR, PIK3R3
	hsa-miR-503-5p	IKBKB, PIK3R1, VEGFA
	hsa-miR-708-5p	AKT1, AKT2
**Toll-like receptor signalling pathway**	hsa-miR-146a-5p	CCL5, CD80, CXCL8, FADD, IL6, IRAK1, NFKB1, RAC1, STAT1, TLR2, TLR4, TRAF6
	hsa-miR-708-5p	AKT1, AKT2, IKBKG

Table S3. Significantly down-regulated TGFβ1-responsive miRNAs associated with glaucoma-related signalling pathways and their gene targets.

KEGG Pathway	miRNA	Gene Targets
**PI3K-Akt signalling pathway**	hsa-miR-15a-5p	AKT3, BCL2, BRCA1, CCND1, CCND2, CCNE1, CHUK, FGF7, MYB, PHLPP1, TP53, VEGFA
	hsa-miR-16-5p	AKT3, BCL2, BRCA1, CCND1, CCND2, CCND3, CCNE1, CDK6, CHUK, FGF2, FGFR1, HGF, IGF1R, KDR, KRAS, MTOR, MYB, RAF1, RPS6KB1, TP53, VEGFA
	hsa-miR-20a-5p	BCL2, BCL2L11, CCND1, CCND2, CDKN1A, ITGB8, KIT, MCL1, MYC, PHLPP2, PPP2R2A, PTEN, RBL2, VEGFA
	hsa-miR-26a-1-3p	GSK3B, PCK1, PTEN
	hsa-miR-26a-5p	CCND2, CCNE1, CCNE2, CDK6, FGF9, GSK3B, HGF, IFNB1, IGF1, IL6, ITGA5, MCL1, NRAS, PIK3CG, PTEN
	hsa-miR-29a-3p	AKT2, AKT3, BCL2, CCND1, CCND2, CDK2, CDK4, CDK6, COL1A2, COL3A1, COL4A1, COL4A2, COL5A2, FOXO3, GSK3B, IFNAR1,

		IGF1, ITGA11, ITGA6, ITGB1, LAMC2, MCL1, MYC, PDGFRB, PIK3R1, PTEN, VEGFA
	hsa-miR-29b-3p	AKT2, AKT3, BCL2, CCND2, CDK6, COL1A1, COL3A1, COL4A1, COL4A2, COL5A2, COL5A3, GSK3B, ITGA6, ITGB1, LAMC1, LAMC2, MCL1, MYC, PDGFA, PDGFB, PDGFC, PDGFRA, PDGFRB, PIK3CG, PIK3R1, PTEN, TCL1A, VEGFA
	hsa-miR-29c-3p	AKT2, AKT3, BCL2, CCND2, CDK6, COL1A1, COL1A2, COL3A1, COL4A1, COL4A2, COL5A2, CREB5, ITGA6, ITGB1, LAMC1, LAMC2, MCL1, PDGFRB, PTEN, VEGFA
	hsa-miR-34a-5p	AKT1, BCL2, CCND1, CCNE2, CDK4, CDK6, CSF1R, IFNB1, IL6R, KIT, MAP2K1, MET, MYB, MYC, PDGFRA, PDGFRB, PIK3CG, TP53
	hsa-miR-34c-5p	BCL2, CCND1, CCNE2, CDK4, CDK6, IL6R, MAP2K1, MET, MYC, PDGFRA, PDGFRB
	hsa-miR-99a-5p	AKT1, FGFR3, IGF1R, MTOR
	hsa-miR-101-3p	CCND1, ITGA3, JAK2, MCL1, MET, MTOR, PIK3CB, RAC1, VEGFA, VEGFC
	hsa-miR-146b-5p	EGFR, IL6, KIT, NFKB1, PDGFRA, TLR4
	hsa-miR-195-5p	BCL2, CCND1, CCND3, CCNE1, CDK4, CDK6, CHUK, FGF2, INSR, KDR, MYB, RAF1, RPS6KB1, VEGFA
	hsa-miR-204-5p	BCL2, CCND1, CCND2, COL3A1, COL5A3, MCL1, MDM2, ITGB3
	hsa-miR-340-5p	AKT1, CCND1, CCND2, CDK6, IL4, KRAS, MDM2, MET
	hsa-miR-378a-3p	CDK6, GRB2, IGF1R, MAPK1, MYC, VEGFA
	hsa-miR-497-5p	BCL2, CCND3, CCNE1, CDK4, EIF4E, IGF1R, IKBKB, MAP2K1, MTOR, RAF1, RPS6KB1
**MAPK signalling pathway**	hsa-miR-15a-5p	AKT3, BDNF, CHUK, CRKL, FGF7, HSPA1B, TP53
	hsa-miR-16-5p	AKT3, BDNF, CHUK, FGF2, FGFR1, KRAS, RAF1, TP53
	hsa-miR-29b-3p	AKT2, AKT3, CDC42, DUSP2, FOS, MYC, PDGFA, PDGFB, PDGFRA, PDGFRB, TGFB1, TGFB2, TGFB3
	hsa-miR-146b-5p	EGFR, NFKB1, PDGFRA, TRAF6
	hsa-miR-378a-3p	GRB2, MAPK1, MYC, TGFB2
	hsa-miR-18a-5p	HIF1A, SMAD2, SMAD3
	hsa-miR-20b-5p	HIF1A, MAPK9, STAT3

**AGE-RAGE signalling pathway in diabetic complications**	hsa-miR-195-5p	CDC42, CHUK, INSR, RAF1
	hsa-miR-204-5p	ALPL, CDC42, EZR, JAK2, MAP2K1, MMP9
	hsa-miR-218-5p	EGFR, IKBKB, MMP2, SP1
	hsa-miR-340-5p	AKT1, RHOA, ROCK1, STAT3
	hsa-miR-497-5p	IKBKB, MAP2K1, RAF1
**Focal adhesion**	hsa-miR-15a-5p	AKT3, BCL2, CCND1, CCND2, CRKL, VEGFA
	hsa-miR-16-5p	AKT3, BCL2, CCND1, CCND2, CCND3, HGF, IGF1R, KDR, RAF1, VEGFA, ZYX
	hsa-miR-20b-5p	AKT3, MAPK9, PTEN, VEGFA
	hsa-miR-26a-1-3p	GSK3B, PTEN
	hsa-miR-29a-3p	AKT2, AKT3, BCL2, CCND1, CCND2, CDC42, COL1A2, COL3A1, COL4A1, COL4A2, COL5A2, GSK3B, IGF1, ITGA11, ITGA6, ITGB1, LAMC2, PDGFRB, PIK3R1, PTEN, VEGFA
	hsa-miR-29b-3p	AKT2, AKT3, BCL2, CCND2, CDC42, COL1A1, COL3A1, COL4A1, COL4A2, COL5A2, COL5A3, GSK3B, ITGA6, ITGB1, LAMC1, LAMC2, PDGFA, PDGFB, PDGFC, PDGFRA, PDGFRB, PIK3CG, PIK3R1, PTEN, VEGFA
	hsa-miR-29c-3p	AKT2, AKT3, BCL2, CCND2, CDC42, COL1A1, COL1A2, COL3A1, COL4A1, COL4A2, COL5A2, ITGA6, ITGB1, LAMC1, LAMC2, PDGFRB, PTEN, VEGFA
	hsa-miR-34a-5p	AKT1, BCL2, CCND1, ERBB2, MAP2K1, MET, PDGFRA, PDGFRB, PIK3CG, PPP1CC, SRC
	hsa-miR-34c-5p	BCL2, CCND1, MAP2K1, MET, PDGFRA, PDGFRB
	hsa-miR-101-3p	CCND1, CTNNB1, ITGA3, MET, PIK3CB, RAC1, RAP1B, RHOA, VEGFA, VEGFC
	hsa-miR-195-5p	BCL2, CCND1, CCND3, CDC42, KDR, RAF1, VEGFA
	hsa-miR-204-5p	BCL2, BIRC2, CCND1, CCND2, COL3A1, ITGB3
	hsa-miR-378a-3p	GRB2, IGF1R, MAPK1, VEGFA
	hsa-miR-497-5p	BCL2, CCND3, IGF1R, MAP2K1, RAF1
**mTOR signalling pathway**	hsa-miR-16-5p	AKT3, MTOR, RICTOR, RPS6KB1, VEGFA
	hsa-miR-19a-3p	AKT1, PIK3CA, PTEN, TNF
	hsa-miR-20b-5p	AKT3, HIF1A, PTEN, VEGFA
	hsa-miR-29a-3p	AKT2, AKT3, IGF1, PIK3R1, PTEN, VEGFA
	hsa-miR-29b-3p	AKT2, AKT3, PIK3CG, PIK3R1, PTEN, VEGFA
	hsa-miR-99a-5p	AKT1, MTOR
	hsa-miR-218-5p	IKBKB, RICTOR, RPS6KA3, RPS6KB1
	hsa-miR-497-5p	EIF4E, IKBKB, MTOR, RPS6KB1

**FoxO signalling pathway**	hsa-miR-15a-5p	AKT3, CCND1, CCND2, CDKN2B, CHUK, FOXO1
	hsa-miR-16-5p	AKT3, CCND1, CCND2, CHUK, IGF1R, KRAS, RAF1
	hsa-miR-18a-5p	ATM, PTEN, SMAD2, SMAD3, SMAD4, STK4, TGFBR2
	hsa-miR-19a-3p	AKT1, BCL2L11, CCND1, IL10, PIK3CA, PTEN, SMAD4, TGFBR2
	hsa-miR-19b-3p	BCL2L11, PRKAA1, PTEN, SMAD4, TGFBR2
	hsa-miR-20a-5p	BCL2L11, CCND1, CCND2, CDKN1A, PTEN, RBL2, SMAD4, STAT3, TGFBR1, TGFBR2
	hsa-miR-20b-5p	AKT3, CDKN1A, MAPK9, PTEN, STAT3
	hsa-miR-26a-1-3p	PCK1, PTEN
	hsa-miR-26a-5p	ATM, CCND2, IGF1, IL6, NRAS, PIK3CG, PTEN, SMAD4
	hsa-miR-29a-3p	AKT2, AKT3, CCND1, CCND2, CDK2, FOXO3, IGF1, PIK3R1, PTEN
	hsa-miR-29b-3p	AKT2, AKT3, CCND2, PIK3CG, PIK3R1, PTEN, STAT3, TGFB1, TGFB2, TGFB3
	hsa-miR-204-5p	CCND1, CCND2, MDM2, TGFBR2
	hsa-miR-340-5p	AKT1, CCND1, CCND2, CCNG2, KRAS, MDM2, SKP2, STAT3
	hsa-miR-378a-3p	GRB2, IGF1R, MAPK1, TGFB2
	hsa-miR-497-5p	IGF1R, IKBKB, MAP2K1, RAF1
**TNF signalling pathway**	hsa-miR-29b-3p	AKT2, AKT3, FOS, MMP9, PIK3CG, PIK3R1, TNFAIP3
**Ras signalling pathway**	hsa-miR-16-5p	AKT3, CHUK, FGF2, FGFR1, HGF, IGF1R, KDR, KRAS, RAF1, VEGFA
	hsa-miR-29b-3p	AKT2, AKT3, CDC42, PDGFA, PDGFB, PDGFC, PDGFRA, PDGFRB, PIK3CG, PIK3R1, VEGFA
	hsa-miR-99a-5p	AKT1, FGFR3, IGF1R
	hsa-miR-101-3p	MET, PIK3CB, RAB5A, RAC1, RAP1B, RHOA, VEGFA, VEGFC
	hsa-miR-146b-5p	EGFR, KIT, NFKB1, PDGFRA
	hsa-miR-195-5p	CDC42, CHUK, FGF2, INSR, KDR, RAF1, VEGFA
	hsa-miR-340-5p	AKT1, KRAS, MET, RHOA
	hsa-miR-378a-3p	GRB2, IGF1R, KSR1, MAPK1, VEGFA
**Neurotrophin signalling pathway**	hsa-miR-15a-5p	AKT3, BCL2, BDNF, CRKL, TP53
	hsa-miR-16-5p	AKT3, ARHGDIA, BCL2, BDNF, KRAS, PRDM4, RAF1, TP53
	hsa-miR-26a-1-3p	GSK3B, PRKCD

	hsa-miR-29a-3p	ABL1, AKT2, AKT3, BCL2, CDC42, FOXO3, GSK3B, PIK3R1
	hsa-miR-29b-3p	AKT2, AKT3, BCL2, CDC42, GSK3B, PIK3CG, PIK3R1
	hsa-miR-146b-5p	IRAK1, NFKB1, TRAF6
	hsa-miR-204-5p	BCL2, IRAK1, NTRK2
	hsa-miR-340-5p	AKT1, KRAS, RHOA
	hsa-miR-497-5p	BCL2, IKBKB, MAP2K1, RAF1
**Wnt signalling pathway**	hsa-miR-16-5p	AXIN2, CCND1, CCND2, CCND3, TP53, WNT3A, WNT4
	hsa-miR-20b-5p	BAMBI, FZD6, MAPK9
	hsa-miR-26a-1-3p	GSK3B, TCF7L2
	hsa-miR-34a-5p	AXIN2, CCND1, FOSL1, LEF1, MYC, SMAD4, TCF7, TP53, WNT1
	hsa-miR-195-5p	BTRC, CCND1, CCND3, NKD1, WNT7A
	hsa-miR-204-5p	CCND1, CCND2, FZD1
	hsa-miR-340-5p	CCND1, CCND2, RHOA
**Hippo signalling pathway**	hsa-miR-16-5p	AXIN2, BIRC5, CCND1, CCND2, CCND3, WNT3A, WNT4, YAP1
	hsa-miR-18a-5p	CCN2, SMAD2, SMAD3, SMAD4, TGFBR2
	hsa-miR-20a-5p	BMPR2, CCND1, CCND2, MYC, PPP2R2A, SMAD4, SMAD7, TGFBR1, TGFBR2
	hsa-miR-26a-1-3p	GSK3B, TCF7L2
	hsa-miR-34a-5p	AXIN2, BIRC5, BMP7, CCND1, GDF5, LEF1, MYC, PPP1CC, SMAD4, SOX2, TCF7, WNT1
	hsa-miR-195-5p	BIRC5, BTRC, CCND1, CCND3, NKD1, SMAD7, WNT7A, YAP1
	hsa-miR-204-5p	BIRC2, CCND1, CCND2, SERPINE1, TGFB2
	hsa-miR-340-5p	CCND1, CCND2, SOX2
	hsa-miR-497-5p	AMOT, BIRC5, BTRC, CCND3, SMAD7, WNT7A
**Apoptosis**	hsa-miR-15a-5p	AKT3, BCL2, CHUK, TP53
	hsa-miR-29b-3p	AKT2, AKT3, BCL2, PIK3CG, PIK3R1
	hsa-miR-146b-5p	IRAK1, NFKB1
	hsa-miR-204-5p	BCL2, BIRC2, IL1B, IL1RAP, IRAK1
**TGF-beta signalling pathway**	hsa-miR-18a-5p	SMAD2, SMAD3, SMAD4, TGFBR2
	hsa-miR-20a-5p	BAMBI, BMPR2, MYC, RBL1, SMAD4, SMAD7, TGFBR1, TGFBR2, ZFYVE9
	hsa-miR-29b-3p	IFNG, MYC, SP1, TGFB1, TGFB2, TGFB3
	hsa-miR-204-5p	TGFBR2
	hsa-miR-335-5p	ID4, MAPK1, MYC, ROCK1, SP1
	hsa-miR-378a-3p	MAPK1, MYC, TGFB2
	hsa-miR-497-5p	RPS6KB1, SMAD7, SMURF1

**Rap1 signalling pathway**	hsa-miR-16-5p	ADORA2A, AKT3, FGF2, FGFR1, HGF, IGF1R, KDR, KRAS, RAF1, VEGFA
	hsa-miR-29b-3p	AKT2, AKT3, CDC42, ITGB1, PDGFA, PDGFB, PDGFC, PDGFRA, PDGFRB, PIK3CG, PIK3R1, VEGFA
	hsa-miR-29c-3p	AKT2, AKT3, CDC42, CTNND1, ITGB1, PDGFRB, TIAM1, VEGFA
	hsa-miR-99a-5p	AKT1, FGFR3, IGF1R
	hsa-miR-101-3p	CTNNB1, MET, PIK3CB, RAC1, RAP1B, RHOA, VEGFA, VEGFC
	hsa-miR-146b-5p	EGFR, KIT, PDGFRA
	hsa-miR-195-5p	CDC42, FGF2, INSR, KDR, RAF1, VEGFA
	hsa-miR-340-5p	AKT1, KRAS, MET, RHOA
**Cell cycle**	hsa-miR-15a-5p	AKT3, BRCA1, CCND1, CCND2, CCNE1, CDC25A, CDKN2B, CHEK1, TP53, WEE1
	hsa-miR-16-5p	AKT3, BIRC5, BRCA1, CCND1, CCND2, CCND3, CCNE1, CDK6, CHEK1, TP53, WEE1
	hsa-miR-20a-5p	CCND1, CCND2, CDKN1A, E2F1, MYC, PPP2R2A, RB1, RBL1, RBL2, UBE2C, WEE1
	hsa-miR-26a-5p	ATM, CCND2, CCNE1, CCNE2, CDC6, CDK6, CHEK1, E2F2, GSK3B, RB1, WEE1
	hsa-miR-34a-5p	AKT1, BIRC5, CCND1, CCNE2, CDK4, CDK6, CDKN2C, E2F1, E2F3, HDAC1, MDM4, MYC, PPP1CC, RAD51, SRC, TP53
	hsa-miR-195-5p	BIRC5, BTRC, CCND1, CCND3, CCNE1, CDC25A, CDK4, CDK6, CHEK1, E2F3, WEE1
	hsa-miR-204-5p	CCND1, CCND2, CDC23, MDM2
	hsa-miR-340-5p	AKT1, CCND1, CCND2, CDK6, MDM2, SKP2
	hsa-miR-497-5p	BIRC5, BTRC, CCND3, CCNE1, CDC25A, CDK4, CHEK1, E2F3, WEE1
**JAK-STAT signalling pathway**	hsa-miR-15a-5p	AKT3, CCND1, CCND2, IFNG, IL10RA
	hsa-miR-16-5p	AKT3, CCND1, CCND2, CCND3, IFNG, IL12B, PIM1, SOCS3
	hsa-miR-19a-3p	AKT1, CCND1, IL10, PIK3CA, SOCS1, SOCS3
	hsa-miR-29b-3p	AKT2, AKT3, CCND2, IFNG, MYC, PIK3CG, PIK3R1, STAT3
	hsa-miR-340-5p	AKT1, CCND1, CCND2, IL4, STAT3
**AMPK signalling pathway**	hsa-miR-29b-3p	AKT2, ELAVL1, HMGCR, PIK3CG, PIK3R1
	hsa-miR-99a-5p	AKT1, MTOR
	hsa-miR-195-5p	CAB39, FASN, INSR, RPS6KB1
	hsa-miR-204-5p	CCND1, EEF2, SCD

**Regulation of actin cytoskeleton**	hsa-miR-29b-3p	CDC42, ITGA6, ITGB1, PDGFA, PDGFB, PDGFC, PDGFRA, PDGFRB, PIK3CG, PIK3R1
	hsa-miR-204-5p	EZR, ITGB3
	hsa-miR-340-5p	KRAS, RHOA, ROCK1
**HIF-1 signalling pathway**	hsa-miR-16-5p	AKT3, BCL2, IFNG, IGF1R, MTOR, RPS6KB1, VEGFA
	hsa-miR-20a-5p	BCL2, CDKN1A, EGLN3, HIF1A, STAT3, VEGFA
	hsa-miR-29b-3p	AKT2, AKT3, BCL2, IFNG, PIK3CG, PIK3R1, STAT3, VEGFA
	hsa-miR-34a-5p	AKT1, BCL2, CYBB, ERBB2, IL6R, LDHA, MAP2K1, PIK3CG
	hsa-miR-99a-5p	AKT1, IGF1R, MTOR
	hsa-miR-146b-5p	EGFR, IL6, NFKB1, TLR4
	hsa-miR-378a-3p	IGF1R, MAPK1, VEGFA
	hsa-miR-204-5p	BCL2, SERPINE1
	hsa-miR-497-5p	BCL2, EIF4E, IGF1R, MAP2K1, MTOR, RPS6KB1
**Relaxin signalling pathway**	hsa-miR-16-5p	AKT1, MAP2K1, VEGFA
	hsa-miR-29a-3p	AKT2, AKT3, COL1A2, COL3A1, COL4A1, COL4A2, PIK3R1, VEGFA
	hsa-miR-29b-3p	AKT2, AKT3, COL1A1, COL3A1, COL4A1, COL4A2, TGFB1, TGFB2, VEGFA
	hsa-miR-29c-3p	AKT2, AKT3, COL1A1, COL1A2, COL3A1, CREB5
	hsa-miR-34a-5p	AKT1, MAP2K1
	hsa-miR-146b-5p	EGFR, MMP2, NFKB1
	hsa-miR-195-5p	RAF1, VEGFA
	hsa-miR-378a-3p	MAPK1, TGFB2
	hsa-miR-1255a	RLN2
**Toll-like receptor signalling pathway**	hsa-miR-146b-5p	IL6, IRAK1, NFKB1, TLR4, TRAF6
**ECM-receptor interaction**	hsa-miR-29a-3p	COL1A2, COL3A1, COL4A1, COL4A2, COL5A2, ITGA11, ITGA6, ITGB1, LAMC2
	hsa-miR-29b-3p	COL1A1, COL3A1, COL4A1, COL4A2, COL5A2, COL5A3, ITGA6, ITGB1, LAMC1, LAMC2
	hsa-miR-29c-3p	COL1A1, COL1A2, COL3A1, COL4A1, COL4A2, COL5A2, ITGA6, ITGB1, LAMC1, LAMC2
	hsa-miR-204-5p	COL3A1, COL5A3, ITGB3
**VEGF signalling pathway**	hsa-miR-16-5p	AKT3, KDR, KRAS, PTGS2, RAF1, VEGFA
	hsa-miR-29a-3p	AKT2, AKT3, CDC42, PIK3R1, VEGFA
	hsa-miR-29b-3p	AKT2, AKT3, CDC42, PIK3CG, PIK3R1, VEGFA
	hsa-miR-101-3p	PIK3CB, PTGS2, RAC1, VEGFA
	hsa-miR-195-5p	CDC42, KDR, RAF1, VEGFA

	hsa-miR-340-5p	AKT1, KRAS
**NF-kappa B signalling pathway**	hsa-miR-18a-5p	ATM, BCL2, TNFAIP3, TNFSF11
	hsa-miR-146b-5p	IRAK1, NFKB1, TLR4, TRAF6
	hsa-miR-195-5p	BCL2, CCL4, CHUK, TAB3

Table S4. Significantly up-regulated TGFβ2-responsive miRNAs associated with glaucoma-related signalling pathways and their gene targets.

KEGG Pathway	miRNA	Gene Targets
**MAPK signalling pathway**	hsa-miR-29b-1-5p	AKT3, JUND, TGFB1
	hsa-miR-143-3p	AKT1, AKT2, BRAF, HRAS, KRAS, MAPK7, NFATC1, NFKB2, TNF
	hsa-miR-181a-5p	DUSP5, DUSP6, FOS, HRAS, KRAS, MAP2K1, MAPK1, NLK, NRAS, PPP3CA, RAP1B, TGFBR1
	hsa-miR-214-3p	ATF4, FGFR1, MAP2K3, MAPK1, MAPK8, MEF2C, NRAS, TP53
**Neurotrophin signalling pathway**	hsa-miR-21-3p	JUN, MAP3K1
	hsa-miR-143-3p	AKT1, AKT2, BCL2, BRAF, HRAS, KRAS, MAPK7
	hsa-miR-181a-5p	BAX, BCL2, HRAS, KRAS, MAP2K1, MAPK1, NRAS, PRKCD, PTPN11, RAP1B
	hsa-miR-181c-5p	BCL2, KRAS, PRKCD, RAP1B
	hsa-miR-214-3p	ATF4, BAX, MAPK1, MAPK8, NRAS, TP53
	hsa-miR-708-5p	AKT1, AKT2, BCL2
**AGE-RAGE signalling pathway in diabetic complications**	hsa-miR-29b-1-5p	SMAD3, STAT3
	hsa-miR-135a-5p	EGFR, FOXO1, JAK2, ROCK1
	hsa-miR-143-3p	AKT1, MMP13, MMP14, MMP2, MMP9
	hsa-miR-145-5p	EGFR, IRS1, MMP14, ROCK1, SMAD2, SMAD3, SP1, STAT1, TIRAP
	hsa-miR-216a-5p	CDC42, JAK2
	hsa-miR-708-5p	AKT1, MMP2, SMAD3
**Wnt signalling pathway**	hsa-miR-21-3p	CCND1, FZD4, JUN
	hsa-miR-145-5p	FZD7, MYC
	hsa-miR-424-5p	CCND1, CCND3, SIAH1, SMAD3
**mTOR signalling pathway**	hsa-miR-143-3p	AKT1, AKT2, BRAF, TNF
	hsa-miR-503-5p	IKBKB, PIK3R1, VEGFA
	hsa-miR-708-5p	AKT1, AKT2
**FoxO signalling pathway**	hsa-miR-21-3p	FASLG, PTEN, STAT3
	hsa-miR-29b-1-5p	AKT3, SMAD3, STAT3, TGFB1
	hsa-miR-143-3p	AKT1, AKT2, BRAF, GABARAPL1, HRAS, IGF1R, KRAS, MDM2
	hsa-miR-145-5p	BNIP3, BRAF, CDKN1A, EGFR, IGF1R, IRS1, IRS2, MDM2, NRAS, SMAD2, SMAD3, TGFB2, TGFBR2
	hsa-miR-181a-5p	ATM, BCL2L11, CDKN1A, CDKN1B, HRAS, KRAS, MAP2K1, MAPK1, NLK, NRAS, PTEN, SIRT1, STAT3, TGFBR1
	hsa-miR-181b-5p	ATM, BCL2L11, IGF1R, MAP2K1, NLK, PTEN, SIRT1

	hsa-miR-181c-5p	KRAS, NLK, PTEN, SIRT1, TGFBR1, TGFBR2
	hsa-miR-503-5p	CCND1, CDKN1A, IGF1R, IKBKB, PIK3R1
	hsa-miR-708-5p	AKT1, AKT2, CCND1, SMAD3
**VEGF signalling pathway**	hsa-miR-143-3p	AKT1, AKT2, HRAS, KRAS, PTGS2
	hsa-miR-181a-5p	HRAS, KRAS, MAP2K1, MAPK1, NRAS, PPP3CA
	hsa-miR-708-5p	AKT1, AKT2
**PI3K-Akt signalling pathway**	hsa-miR-21-3p	CCND1, CDK6, ITGA6, MCL1
	hsa-miR-29b-1-5p	AKT3, COL1A1, COL3A1, COL5A1, JAK3
	hsa-miR-99b-5p	IGF1R, MTOR
	hsa-miR-143-3p	AKT1, AKT2, BCL2, COL3A1, HRAS, IGF1R, ITGB1, ITGB4, KRAS, MDM2, TLR2
	hsa-miR-145-5p	COL1A2, IGF1R, IRS1, ITGA3, MYC, THBS1
	hsa-miR-181a-5p	BCL2, BCL2L11, CDKN1A, CDKN1B, DDIT4, HRAS, KRAS, MAP2K1, MAPK1, MCL1, NRAS, PHLPP2, PTEN
	hsa-miR-181b-5p	BCL2, BCL2L11, CREB1, IGF1R, MAP2K1, MCL1, PTEN, SPP1, TCL1A
	hsa-miR-214-3p	ATF4, BCL2L11, CDK6, FGFR1, MAPK1, NRAS, PTEN, TP53
	hsa-miR-214-5p	CDK6, IGF1R, TP53
	hsa-miR-424-5p	CCND1, CCND3, CCNE1, CDK6, FGF2, FGFR1, MAP2K1, MYB
	hsa-miR-503-5p	BCL2, CCND1, CCND3, CCNE1, CCNE2, CDKN1A, FGF2, FGF8, FGFR1, IGF1R, IKBKB, MYB, PIK3R1, VEGFA
	hsa-miR-708-5p	AKT1, AKT2, BCL2, CCND1, IKBKG
**Focal adhesion**	hsa-miR-21-3p	CCND1, ITGA6, JUN, PPP1CC
	hsa-miR-29b-1-5p	AKT3, COL1A1, COL3A1, COL5A1
	hsa-miR-143-3p	AKT1, AKT2, BCL2, BRAF, COL3A1, HRAS, IGF1R, ITGB1, ITGB4
	hsa-miR-145-5p	ACTB, ACTG1, COL1A2, IGF1R, ITGA3, PAK1, THBS1
	hsa-miR-181a-5p	BCL2, CTNNB1, HRAS, MAP2K1, MAPK1, PTEN, RAP1B, XIAP
	hsa-miR-181b-5p	BCL2, IGF1R, MAP2K1, PTEN, RAP1B, SPP1, XIAP
	hsa-miR-503-5p	BCL2, CCND1, CCND3, IGF1R, PIK3R1, VEGFA
	hsa-miR-708-5p	AKT1, AKT2, BCL2, CCND1
**Hippo signalling pathway**	hsa-miR-21-3p	CCND1, FZD4, PPP1CC
	hsa-miR-29b-1-5p	SMAD3, TGFB1
	hsa-miR-145-5p	ACTB, ACTG1, CTGF, FZD7, MYC, SERPINE1
	hsa-miR-181c-5p	BMPR2, LATS2, SAV1, TGFBR1, TGFBR2
	hsa-miR-424-5p	CCND1, CCND3, SMAD3, SMAD7

	hsa-miR-708-5p	BIRC5, CCND1, SMAD3
**TNF signalling pathway**	hsa-miR-143-3p	AKT1, AKT2, MMP14, MMP9, PTGS2, TNF
	hsa-miR-214-3p	ATF4, CCL5, JAG1, MAP2K3, MAPK1, MAPK8
	hsa-miR-708-5p	AKT1, AKT2, IKBKG
**Relaxin signalling pathway**	hsa-miR-29b-1-5p	AKT3, COL3A1
	hsa-miR-135a-5p	EGFR
	hsa-miR-143-3p	COL3A1, HRAS
	hsa-miR-145-5p	EGFR, NRAS, SMAD2
	hsa-miR-181a-5p	FOS, HRAS, MAP2K1, NRAS
	hsa-miR-181b-5p	MAP2K1
	hsa-miR-214-3p	NRAS
	hsa-miR-424-5p	MAP2K1
	hsa-miR-503-5p	VEGFA
**Ras signalling pathway**	hsa-miR-181a-5p	HRAS, KRAS, MAP2K1, MAPK1, NRAS, PTPN11, RALA, RAP1B, RASSF1
	hsa-miR-214-5p	IGF1R, RASSF5
	hsa-miR-503-5p	FGF2, FGF8, FGFR1, IGF1R, IKBKB, PIK3R1, VEGFA
**Rap1 signalling pathway**	hsa-miR-143-3p	AKT1, AKT2, BRAF, HRAS, IGF1R, ITGB1, KRAS
	hsa-miR-214-5p	IGF1R, RASSF5
	hsa-miR-503-5p	FGF2, FGF8, FGFR1, IGF1R, PIK3R1, VEGFA
**Apoptosis**	hsa-miR-143-3p	AKT1, AKT2, BCL2, TNF
	hsa-miR-181a-5p	ATM, BAX, BCL2, PPP3CA, XIAP
	hsa-miR-503-5p	BCL2, IKBKB, PIK3R1
	hsa-miR-708-5p	AKT1, AKT2, BCL2, IKBKG
**Regulation of actin cytoskeleton**	hsa-miR-21-3p	ITGA6, PPP1CC
	hsa-miR-135a-5p	APC, EGFR, PTK2, ROCK1, ROCK2
	hsa-miR-143-3p	BRAF, HRAS, ITGB1, ITGB4, KRAS, LIMK1
	hsa-miR-145-5p	ACTB, ACTG1, ITGA3, PAK1
	hsa-miR-503-5p	FGF2, FGF8, FGFR1, PIK3R1
**Notch signalling pathway**	hsa-miR-199b-5p	HES1, JAG1
**Toll-like receptor signalling pathway**	hsa-miR-143-3p	AKT1, AKT2, TLR2, TNF
	hsa-miR-708-5p	AKT1, AKT2, IKBKG
**NF-kappa B signalling pathway**	hsa-miR-143-3p	BCL2, NFKB2, PTGS2, TNF
	hsa-miR-503-5p	BCL2, CD40, IKBKB, TNFRSF11A
	hsa-miR-708-5p	BCL2, IKBKG, PARP1
**HIF-1 signalling pathway**	hsa-miR-21-3p	STAT3
	hsa-miR-29b-1-5p	AKT3, STAT3
	hsa-miR-99b-5p	IGF1R, MTOR
	hsa-miR-143-3p	AKT1, AKT2, BCL2, HK2, IGF1R, SERPINE1

	hsa-miR-145-5p	HK2, IGF1R, SERPINE1, TIMP1
	hsa-miR-181a-5p	BCL2, CDKN1A, CDKN1B, IFNG, MAP2K1, MAPK1, STAT3, TIMP1
	hsa-miR-503-5p	BCL2, CDKN1A, IGF1R, PIK3R1, VEGFA
	hsa-miR-708-5p	AKT1, AKT2, BCL2
**JAK-STAT signalling pathway**	hsa-miR-29b-1-5p	AKT3, JAK3, STAT3
	hsa-miR-708-5p	AKT1, AKT2, CCND1, CNTFR

Table S5. Significantly down-regulated TGFβ2-responsive miRNAs associated with glaucoma-related signalling pathways and their gene targets.

KEGG Pathway	miRNA	Gene Targets
**Neurotrophin signalling pathway**	hsa-miR-15a-3p	AKT3, BCL2, BDNF, CRKL, TP53
	hsa-miR-26a-1-3p	GSK3B, PRKCD
	hsa-miR-29b-3p	AKT2, AKT3, BCL2, CDC42, GSK3B, PIK3CG, PIK3R1
	hsa-miR-204-5p	BCL2, IRAK1, NTRK2
	hsa-miR-302b-3p	AKT1, AKT2, MAP3K1
**Hippo signalling pathway**	hsa-miR-26a-1-3p	GSK3B, TCF7L2
	hsa-miR-29b-3p	ACTB, CCND1, CCND2, DVL3, FZD5, SMAD2, TGFB2
	hsa-miR-200a-3p	AREG, CTNNB1, SMAD2, SMAD3, TCF7L1, TGFB2, YAP1
	hsa-miR-204-5p	BIRC2, CCND1, CCND2, SERPINE1, TGFB2
	hsa-miR-324-3p	DVL2, WNT2B, WNT9B
**Wnt signalling pathway**	hsa-miR-20b-5p	BAMBI, FZD6, MAPK9
	hsa-miR-26a-1-3p	GSK3B, TCF7L2
	hsa-miR-29b-3p	CCND1, CCND2, DVL3, FZD5, JUN, SIAH1, TP53
	hsa-miR-204-5p	CCND1, CCND2, EZD1
	hsa-miR-324-3p	CREBBP, DVL2, WNT2B, WNT9B
	hsa-miR-582-3p	AXIN2, SFRP1
**Focal adhesion**	hsa-miR-15a-3p	AKT3, BCL2, CCND1, CCND2, CRKL, VEGFA
	hsa-miR-20b-5p	AKT3, MAPK9, PTEN, VEGFA
	hsa-miR-26a-1-3p	GSK3B, PTEN
	hsa-miR-29b-3p	AKT2, AKT3, BCL2, CCND2, CDC42, COL1A1, COL3A1, COL4A1, COL4A2, COL5A2, COL5A3, GSK3B, ITGA6, ITGB1, LAMC1, LAMC2, PDGFA, PDGFB, PDGFC, PDGFRA, PDGFRB, PIK3CG, PIK3R1, PTEN, VEGFA
	hsa-miR-204-5p	BCL2, BIRC2, CCND1, CCND2, COL3A1, ITGB3
	hsa-miR-302b-3p	AKT1, AKT2, CCND2, EGFR

**PI3K-Akt signalling pathway**	hsa-miR-15a-3p	AKT3, BCL2, BRCA1, CCND1, CCND2, CCNE1, CHUK, FGF7, MYB, PHLPP1, TP53, VEGFA
	hsa-miR-20b-5p	AKT3, BRCA1, CDKN1A, PTEN, VEGFA
	hsa-miR-26a-1-3p	GSK3B, PCK1, PTEN
	hsa-miR-29b-3p	AKT2, AKT3, BCL2, CCND2, CDK6, COL1A1, COL3A1, COL4A1, COL4A2, COL5A2, COL5A3, GSK3B, ITGA6, ITGB1, LAMC1, LAMC2, MCL1, MYC, PDGFA, PDGFB, PDGFC, PDGFRA, PDGFRB, PIK3CG, PIK3R1, PTEN, TCL1A, VEGFA
	hsa-miR-204-5p	BCL2, CCND1, CCND2, COL3A1, COL5A3, ITGB3, MCL1, MDM2
	hsa-miR-302b-3p	AKT1, AKT2, CCND2, CDK2, EGFR, MCL1
	hsa-miR-582-3p	CASP9, CREB1, MCL1
**Relaxin signalling pathway**	hsa-miR-29b-3p	AKT2, COL4A2, TGFB1
	hsa-miR-138-5p	AKT1
	hsa-miR-200a-3p	EGFR, SHC1
	hsa-miR-204-5p	MMP9
	hsa-miR-218-5p	EGFR, MMP2
	hsa-miR-302b-3p	AKT1, AKT2, EGFR
	hsa-miR-452-3p	KRAS
**Apoptosis**	hsa-miR-15a-3p	AKT3, BCL2, CHUK, TP53
	hsa-miR-29b-3p	AKT2, AKT3, BCL2, PIK3CG, PIK3R1
	hsa-miR-204-5p	BCL2, BIRC2, IL1B, IL1RP8, IRAK1
	hsa-miR-302b-3p	AKT1, AKT2
**AGE-RAGE signalling pathway in diabetic complications**	hsa-miR-20b-5p	HIF1A, MAPK9, STAT3
	hsa-miR-138-5p	AKT1, CASP3, HIF1A, NFKB1
	hsa-miR-200a-3p	EGFR, MAPK14, SHC1, SMAD2, SMAD3
	hsa-miR-204-5p	ALPL, CDC42, EZR, JAK2, MAP2K1, MMP9
	hsa-miR-218-5p	EGFR, IKBKB, MMP2, SP1
	hsa-miR-302b-3p	AKT1, EGFR
	hsa-miR-330-3p	CDC42, SP1
**Cell cycle**	hsa-miR-15a-3p	AKT3, BRCA1, CCND1, CCND2, CCNE1, CDC25A, CDKN2B, CHEK1, TP53, WEE1
	hsa-miR-204-5p	CCND1, CCND2, CDC23, MDM2
	hsa-miR-302b-3p	AKT1, AKT2, CCND2, CDK2, E2F1
	hsa-miR-760	CSNK2A1, HIST1H2AD
**mTOR signalling pathway**	hsa-miR-20b-5p	AKT3, HIF1A, PTEN, VEGFA
	hsa-miR-29b-3p	AKT2, AKT3, PIK3CG, PIK3R1, PTEN, VEGFA
	hsa-miR-218-5p	IKBKB, RICTOR, RPS6KA3, RPS6KB1
	hsa-miR-302b-3p	AKT1, AKT2
	hsa-miR-29b-3p	AKT2, ELAVL1, HMGCR, PIK3CG, PIK3R1

**AMPK signalling pathway**	hsa-miR-204-5p	CCND1, EEF2, SCD
	hsa-miR-302b-3p	AKT1, AKT2
**MAPK signalling pathway**	hsa-miR-15a-3p	AKT3, BDNF, CHUK, CRKL, FGF7, HSPA1B, TP53
	hsa-miR-29b-3p	AKT2, AKT3, CDC42, DUSP2, FOS, MYC, PDGFA, PDGFB, PDGFRA, PDGFRB, TGFB1, TGFB2, TGFB3
	hsa-miR-302b-3p	AKT1, AKT2, EGFR, MAP3K1, TGFBR2
**FoxO signalling pathway**	hsa-miR-15a-3p	AKT3, CCND1, CCND2, CDKN2B, CHUK, FOXO1
	hsa-miR-20b-5p	AKT3, CDKN1A, MAPK9, PTEN, STAT3
	hsa-miR-26a-1-3p	PCK1, PTEN
	hsa-miR-29b-3p	AKT2, AKT3, CCND2, PIK3CG, PIK3R1, PTEN, STAT3, TGFB1, TGFB2, TGFB3
	hsa-miR-200a-3p	EGFR, GRB2, MAPK14, PTEN, SMAD2, SMAD3, TGFB2
	hsa-miR-204-5p	CCND1, CCND2, MDM2, TGFBR2
	hsa-miR-302b-3p	AKT1, AKT2, CCND2, CDK2, EGFR, TGFBR2
	hsa-miR-452-3p	CDKN1B, KRAS
**VEGF signalling pathway**	hsa-miR-29b-3p	AKT2, AKT3, CDC42, PIK3CG, PIK3R1, VEGFA
	hsa-miR-302b-3p	AKT1, AKT2
**HIF-1 signalling pathway**	hsa-miR-20b-5p	AKT3, CDKN1A, HIF1A, STAT3, VEGFA
	hsa-miR-29b-3p	AKT2, AKT3, BCL2, IFNG, PIK3CG, PIK3R1, STAT3, VEGFA
	hsa-miR-204-5p	BCL2, SERPINE1
	hsa-miR-302b-3p	AKT1, AKT2, EGFR

Table S6. Significantly down-regulated TGFβ2-responsive miRNAs associated with glaucoma-related signalling pathways and their gene targets.

KEGG Pathway	miRNA	Gene Targets
**Neurotrophin signalling pathway**	hsa-miR-15a-3p	AKT3, BCL2, BDNF, CRKL, TP53
	hsa-miR-26a-1-3p	GSK3B, PRKCD
	hsa-miR-29b-3p	AKT2, AKT3, BCL2, CDC42, GSK3B, PIK3CG, PIK3R1
	hsa-miR-204-5p	BCL2, IRAK1, NTRK2
	hsa-miR-302b-3p	AKT1, AKT2, MAP3K1
**Hippo signalling pathway**	hsa-miR-26a-1-3p	GSK3B, TCF7L2
	hsa-miR-29b-3p	ACTB, CCND1, CCND2, DVL3, FZD5, SMAD2, TGFB2
	hsa-miR-200a-3p	AREG, CTNNB1, SMAD2, SMAD3, TCF7L1, TGFB2, YAP1
	hsa-miR-204-5p	BIRC2, CCND1, CCND2, SERPINE1, TGFB2
	hsa-miR-324-3p	DVL2, WNT2B, WNT9B
**Wnt signalling pathway**	hsa-miR-20b-5p	BAMBI, FZD6, MAPK9
	hsa-miR-26a-1-3p	GSK3B, TCF7L2
	hsa-miR-29b-3p	CCND1, CCND2, DVL3, FZD5, JUN, SIAH1, TP53
	hsa-miR-204-5p	CCND1, CCND2, EZD1
	hsa-miR-324-3p	CREBBP, DVL2, WNT2B, WNT9B
	hsa-miR-582-3p	AXIN2, SFRP1
**Focal adhesion**	hsa-miR-15a-3p	AKT3, BCL2, CCND1, CCND2, CRKL, VEGFA
	hsa-miR-20b-5p	AKT3, MAPK9, PTEN, VEGFA
	hsa-miR-26a-1-3p	GSK3B, PTEN
	hsa-miR-29b-3p	AKT2, AKT3, BCL2, CCND2, CDC42, COL1A1, COL3A1, COL4A1, COL4A2, COL5A2, COL5A3, GSK3B, ITGA6, ITGB1, LAMC1, LAMC2, PDGFA, PDGFB, PDGFC, PDGFRA, PDGFRB, PIK3CG, PIK3R1, PTEN, VEGFA
	hsa-miR-204-5p	BCL2, BIRC2, CCND1, CCND2, COL3A1, ITGB3
	hsa-miR-302b-3p	AKT1, AKT2, CCND2, EGFR
**PI3K-Akt signalling pathway**	hsa-miR-15a-3p	AKT3, BCL2, BRCA1, CCND1, CCND2, CCNE1, CHUK, FGF7, MYB, PHLPP1, TP53, VEGFA
	hsa-miR-20b-5p	AKT3, BRCA1, CDKN1A, PTEN, VEGFA
	hsa-miR-26a-1-3p	GSK3B, PCK1, PTEN
	hsa-miR-29b-3p	AKT2, AKT3, BCL2, CCND2, CDK6, COL1A1, COL3A1, COL4A1, COL4A2, COL5A2, COL5A3, GSK3B, ITGA6, ITGB1, LAMC1, LAMC2, MCL1, MYC, PDGFA, PDGFB, PDGFC, PDGFRA, PDGFRB, PIK3CG, PIK3R1, PTEN, TCL1A, VEGFA

	hsa-miR-204-5p	BCL2, CCND1, CCND2, COL3A1, COL5A3, ITGB3, MCL1, MDM2
	hsa-miR-302b-3p	AKT1, AKT2, CCND2, CDK2, EGFR, MCL1
	hsa-miR-582-3p	CASP9, CREB1, MCL1
**Relaxin signalling pathway**	hsa-miR-29b-3p	AKT2, COL4A2, TGFB1
	hsa-miR-138-5p	AKT1
	hsa-miR-200a-3p	EGFR, SHC1
	hsa-miR-204-5p	MMP9
	hsa-miR-218-5p	EGFR, MMP2
	hsa-miR-302b-3p	AKT1, AKT2, EGFR
	hsa-miR-452-3p	KRAS
**Apoptosis**	hsa-miR-15a-3p	AKT3, BCL2, CHUK, TP53
	hsa-miR-29b-3p	AKT2, AKT3, BCL2, PIK3CG, PIK3R1
	hsa-miR-204-5p	BCL2, BIRC2, IL1B, IL1RP8, IRAK1
	hsa-miR-302b-3p	AKT1, AKT2
**AGE-RAGE signalling pathway in diabetic complications**	hsa-miR-20b-5p	HIF1A, MAPK9, STAT3
	hsa-miR-138-5p	AKT1, CASP3, HIF1A, NFKB1
	hsa-miR-200a-3p	EGFR, MAPK14, SHC1, SMAD2, SMAD3
	hsa-miR-204-5p	ALPL, CDC42, EZR, JAK2, MAP2K1, MMP9
	hsa-miR-218-5p	EGFR, IKBKB, MMP2, SP1
	hsa-miR-302b-3p	AKT1, EGFR
	hsa-miR-330-3p	CDC42, SP1
**Cell cycle**	hsa-miR-15a-3p	AKT3, BRCA1, CCND1, CCND2, CCNE1, CDC25A, CDKN2B, CHEK1, TP53, WEE1
	hsa-miR-204-5p	CCND1, CCND2, CDC23, MDM2
	hsa-miR-302b-3p	AKT1, AKT2, CCND2, CDK2, E2F1
	hsa-miR-760	CSNK2A1, HIST1H2AD
**mTOR signalling pathway**	hsa-miR-20b-5p	AKT3, HIF1A, PTEN, VEGFA
	hsa-miR-29b-3p	AKT2, AKT3, PIK3CG, PIK3R1, PTEN, VEGFA
	hsa-miR-218-5p	IKBKB, RICTOR, RPS6KA3, RPS6KB1
	hsa-miR-302b-3p	AKT1, AKT2
**AMPK signalling pathway**	hsa-miR-29b-3p	AKT2, ELAVL1, HMGCR, PIK3CG, PIK3R1
	hsa-miR-204-5p	CCND1, EEF2, SCD
	hsa-miR-302b-3p	AKT1, AKT2
**MAPK signalling pathway**	hsa-miR-15a-3p	AKT3, BDNF, CHUK, CRKL, FGF7, HSPA1B, TP53
	hsa-miR-29b-3p	AKT2, AKT3, CDC42, DUSP2, FOS, MYC, PDGFA, PDGFB, PDGFRA, PDGFRB, TGFB1, TGFB2, TGFB3
	hsa-miR-302b-3p	AKT1, AKT2, EGFR, MAP3K1, TGFBR2
**FoxO signalling pathway**	hsa-miR-15a-3p	AKT3, CCND1, CCND2, CDKN2B, CHUK, FOXO1
	hsa-miR-20b-5p	AKT3, CDKN1A, MAPK9, PTEN, STAT3
	hsa-miR-26a-1-3p	PCK1, PTEN

	hsa-miR-29b-3p	AKT2, AKT3, CCND2, PIK3CG, PIK3R1, PTEN, STAT3, TGFB1, TGFB2, TGFB3
	hsa-miR-200a-3p	EGFR, GRB2, MAPK14, PTEN, SMAD2, SMAD3, TGFB2
	hsa-miR-204-5p	CCND1, CCND2, MDM2, TGFBR2
	hsa-miR-302b-3p	AKT1, AKT2, CCND2, CDK2, EGFR, TGFBR2
	hsa-miR-452-3p	CDKN1B, KRAS
**VEGF signalling pathway**	hsa-miR-29b-3p	AKT2, AKT3, CDC42, PIK3CG, PIK3R1, VEGFA
	hsa-miR-302b-3p	AKT1, AKT2
**HIF-1 signalling pathway**	hsa-miR-20b-5p	AKT3, CDKN1A, HIF1A, STAT3, VEGFA
	hsa-miR-29b-3p	AKT2, AKT3, BCL2, IFNG, PIK3CG, PIK3R1, STAT3, VEGFA
	hsa-miR-204-5p	BCL2, SERPINE1
	hsa-miR-302b-3p	AKT1, AKT2, EGFR
